# Key Factors for a One-Pot Enzyme Cascade Synthesis of High Molecular Weight Hyaluronic Acid

**DOI:** 10.3390/ijms20225664

**Published:** 2019-11-12

**Authors:** Johannes Gottschalk, Henning Zaun, Anna Eisele, Jürgen Kuballa, Lothar Elling

**Affiliations:** 1 Laboratory for Biomaterials, Institute of Biotechnology and Helmholtz-Institute for Biomedical Engineering, RWTH Aachen University, Pauwelsstraße 20, 52074 Aachen, Germany; j.gottschalk@biotec.rwth-aachen.de (J.G.); a.eisele@biotec.rwth-aachen.de (A.E.); 2 Research and Development Department, GALAB Laboratories GmbH, Am Schleusengraben 7, 21029 Hamburg, Germany; henning.zaun@galab.de (H.Z.); juergen.kuballa@galab.de (J.K.)

**Keywords:** hyaluronic acid, in vitro synthesis, one-pot multi-enzyme, optimization, enzyme cascade

## Abstract

In the last decades, interest in medical or cosmetic applications of hyaluronic acid (HA) has increased. Size and dispersity are key characteristics of biological function. In contrast to extraction from animal tissue or bacterial fermentation, enzymatic in vitro synthesis is the choice to produce defined HA. Here we present a one-pot enzyme cascade with six enzymes for the synthesis of HA from the cheap monosaccharides glucuronic acid (GlcA) and *N*-acetylglucosamine (GlcNAc). The combination of two enzyme modules, providing the precursors UDP–GlcA and UDP–GlcNAc, respectively, with hyaluronan synthase from *Pasteurella multocida* (PmHAS), was optimized to meet the kinetic requirements of PmHAS for high HA productivity and molecular weight. The Mg^2+^ concentration and the pH value were found as key factors. The HA product can be tailored by different conditions: 25 mM Mg^2+^ and 2-[4-(2-hydroxyethyl)piperazin-1-yl]ethanesulfonic acid (HEPES)-NaOH pH 8 result into an HA product with high *M*_w_ HA (1.55 MDa) and low dispersity (1.05). Whereas with 15 mM Mg^2+^ and HEPES–NaOH pH 8.5, we reached the highest HA concentration (2.7 g/L) with a yield of 86.3%. Our comprehensive data set lays the basis for larger scale enzymatic HA synthesis.

## 1. Introduction and Motivation

Hyaluronic acid (HA) is a natural, non-sulfated, linear polymer consisting of repeating disaccharide units of [-3)GlcNAc(β1-4)GlcA(β1-]_n_. In contrast to other glycosaminoglycans, HA is a polysaccharide with a size of up to 10^8^ Da. Due to the anionic character, HA is able to bind large amounts of water, resulting in a viscoelastic gel [1,2,3]. HA occurs in many species. In humans, HA forms are found in the extracellular matrix of connective tissues [3]. Some pathogenic bacteria (*Pasteurella multocida* and *Streptococcus* strains) camouflage themselves with HA capsules to evade the host´s immune reaction [4]. Because of the unique rheological behavior and the non-immunogenic feature, HA is widely used for medical and cosmetic applications such as drug/cosmetic agents, ophthalmic surgery, and tissue engineering [5,6,7,8,9,10]. With the increasing demand for HA, the market is expected to develop from USD 7.2 billion in 2016 to USD 15.5 billion in 2025 [11].

Current industrial production of high molecular weight HA is based on harsh extraction from rooster combs or bacterial fermentation with *Streptococcus*. Contaminations with bird proteins and exotoxins can cause allergic reactions or infections. These processes result in a highly dispersed HA product, which affects the biological properties of HA. Sometimes long and short HA chains have even counteractive effects, making highly disperse products less predictable for medical uses [12,13,14,15]. Fermentation processes with genetically modified microbial strains aiming for high HA titer and low dispersity are under development [12,16].

Enzymatic in vitro synthesis of HA with low dispersity was accomplished with a soluble class 2 hyaluronan synthase (PmHAS) from *P. multocida* [15]. PmHAS binds the nucleotide sugar substrates UDP–GlcA and UDP–GlcNAc at two active sites, respectively, for HA polymerization [17,18,19,20,21,22]. However, HA in vitro production is limited by the availability and high consumption of the expensive substrates UDP–GlcA and UDP–GlcNAc. In this respect, in situ generations of both nucleotide sugars were coupled to PmHAS in a one-pot synthesis of HA reaching a molecular weight between 0.02 and 0.5 MDa with 70% yield (1.4 g/L) [23]. For UDP–GlcA production, glucuronic acid kinase (AtGlcAK) and UDP–sugar pyrophosphorylase (AtUSP) from *Arabidopsis thaliana*, as well as pyrophosphatase (PmPpA) from *P. multocida* are proven candidates [24,25,26,27,28,29,30]. For UDP–GlcNAc, GlcNAc-1-phosphate kinase (BlNahK) from *Bifidobacterium longum* and UDP–GlcNAc pyrophosphorylases from *Streptococcus zooepidemicus* (SzGlmU) or *Campylobacter jejuni* (CjGlmU) as well as PmPpA were successfully applied [23,31,32,33,34]. We recently demonstrated the in vitro one-pot synthesis of HA from sucrose and GlcNAc with in situ regeneration of UDP–GlcA. We obtained HA with a molecular weight of 2 MDa with a low dispersity (1.02) and HA titer of 4 g/L after 8 h. We showed that substrate inhibition by UDP–GlcA and a high *K*_m_ value for UDP–GlcNAc afford a favorable UDP–sugar ratio for the production of HA by PmHAS. In addition, in the presence of Mg^2+^ or Mn^2+^, K^+^ was found to enhance the polymerization rate of PmHAS [31].

**Scheme 1:**
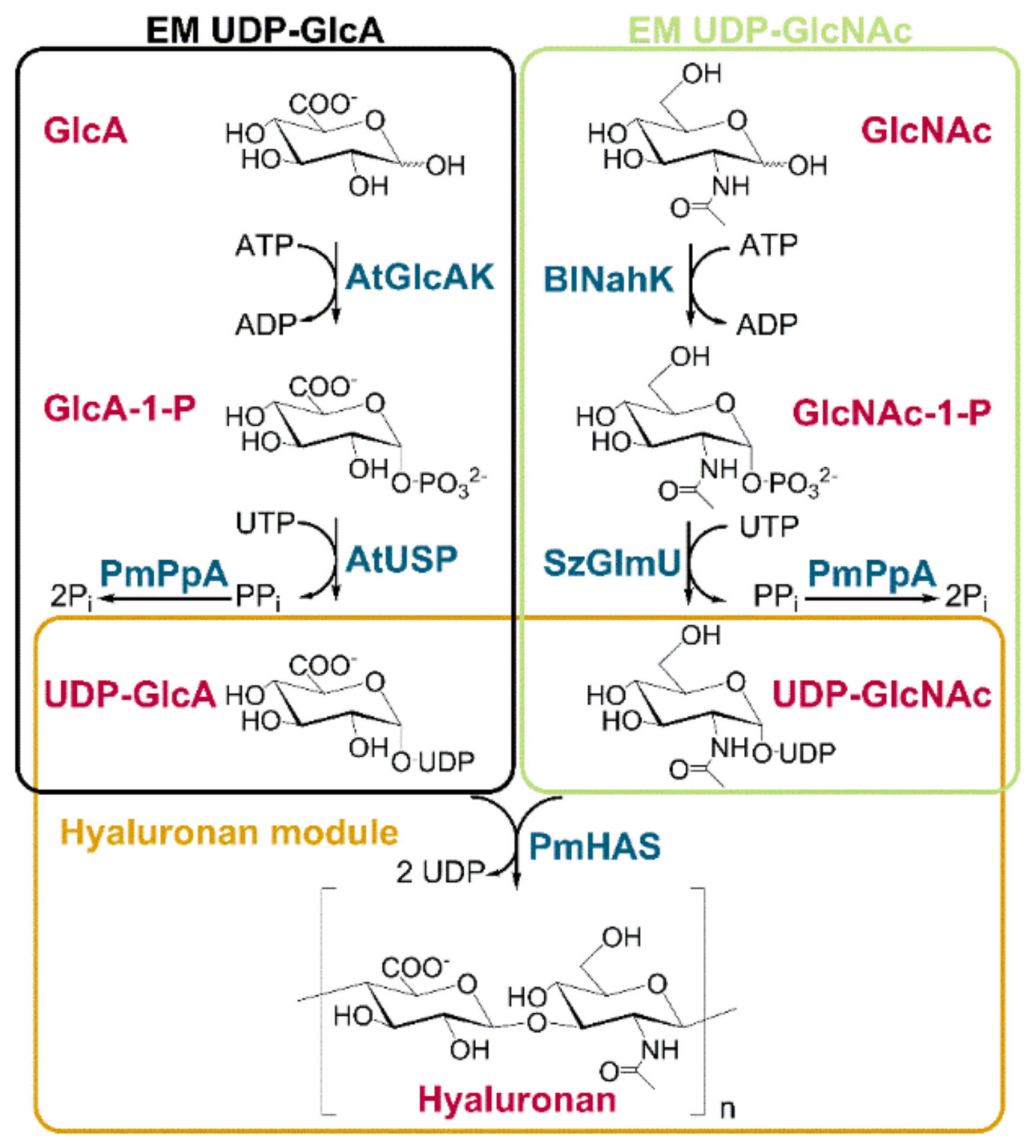
Enzyme cascade for one-pot hyaluronic acid synthesis. The one-pot synthesis is accomplished by three enzyme modules (EM): UDP–GlcA, UDP–GlcNAc, and hyaluronan module. The EM UDP–GlcA contains the enzymes AtGlcAK, AtUSP (both *Arabidopsis thaliana*), and PmPpA (*Pasteurella multocida*). The EM UDP–GlcNAc contains the enzymes BlNahK (*Bifidobacterium longum*), SzGlmU (*Streptococcus zooepidemicus*), and PmPpA. In the hyaluronan module PmHAS (*P. multocida*) polymerizes the HA chain using UDP–GlcA and UDP–GlcNAc as donor substrates.

In order to circumvent the cofactor regeneration of NAD^+^ for UDP–GlcA synthesis, we aimed in the present work for an optimized one-pot synthesis of HA with six recombinant enzymes (Scheme 1). The one-pot synthesis contains three enzyme modules (EM): UDP–GlcA-, UDP–GlcNAc-, and hyaluronan-module. In the EM UDP–GlcA and EM UDP–GlcNAc, AtGlcAK and BlNahK phosphorylate the monosaccharides GlcA and GlcNAc, respectively, to the corresponding sugar-1-phosphates with ATP consumption. AtUSP and SzGlmU convert them to UDP–GlcA and UDP–GlcNAc, respectively, using uridine triphosphate (UTP). PmPpA hydrolyzes the AtUSP and SzGlmU inhibiting byproduct pyrophosphate (PP_i_). Finally, PmHAS utilizes the nucleotide sugars for HA chain polymerization. As a straightforward development, we aimed to optimize and control this enzyme cascade for high yields, high HA concentration and high molecular weight of HA. To investigate multiple parameters for optimization of each enzyme module (EM) we analyzed enzyme reactions by high-throughput multiplexed capillary electrophoresis (MP-CE) monitoring nucleotides and nucleotide sugars [35].

## 2. Results and Discussion

### 2.1. Enzyme Production

The recombinant enzymes were produced in *E. coli* BL21 (DE3) and purified by immobilized metal affinity chromatography (IMAC). The results of the sodium dodecyl sulfate polyacrylamide gel electrophoresis (SDS-PAGE) and Western blot analyses are summarized in the supplemental data (Figure S1).

### 2.2. Characterization of AtGlcAK, AtUSP, and PmPpA in the EM UDP–GlcA

We already analyzed the EM UDP–GlcNAc in our previous study [31]. Therefore, we focused in this study on the EM UDP–GlcA. Especially the enzymes AtGlcAK, AtUSP, and PmPpA were investigated for their kinetics (Table 1), optimal pH value and temperature as well as metal ion dependency (Figures S2–S4).

AtGlcAK was inhibited by ATP concentrations higher than 10 mM (Table 1 and Figure S2A,B) resulting in different kinetic constants compared to published data [23,29,30]. The optimal pH value and temperature is 7.5–9 °C, and 35–45 °C, respectively (Figure S2C,D). The temperature optimum corresponds to a prior study by Pieslinger et al., while we detected a broader pH optimum [30]. Mg^2+^ concentrations should be at least equimolar to the optimal ATP concentration of 5 mM to reach high specific enzyme activity (Figure S2E,F). For AtUSP, kinetic constants (Table 1 and (Figure S3A,B) were similar to published data [23,25,27,36]. The highest specific activity was found at a temperature of 40–45 °C (Figure S3C) and at a pH value of 8.0–8.5 (Figure S3C). Excess of Mg^2+^ over the UTP concentration results in high specific activity (Figure S3E). For PmPpA, a substrate concentration higher than 5 mM PP_i_ strongly inhibits the enzyme activity (Table 1 and Figure S4A). The enzyme has a temperature optimum at 40 °C (Figure S4B), a pH optimum at 8.5–9 (Figure S4D), and at least a three-fold Mg^2+^ excess to the PP_i_ concentration leads to high enzyme activities (Figure S4C).

### 2.3. Characterization of the EM UDP–GlcA

**Figure 1:**
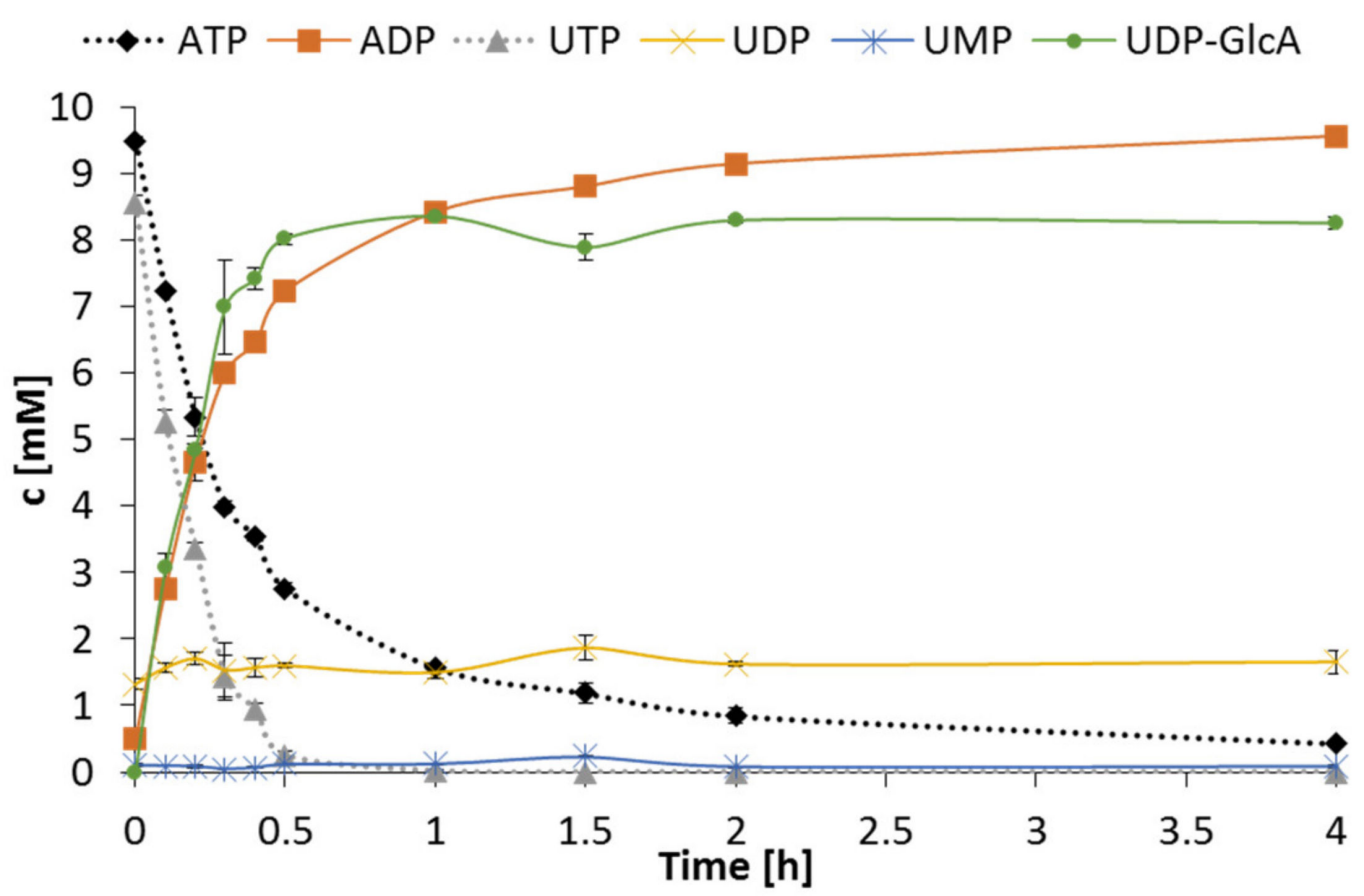
Synthesis of UDP–GlcA with AtGlcAK, AtUSP, and PmPpA. One-pot synthesis was performed under the following conditions: 100 mM 2-[4-(2-hydroxyethyl)piperazin-1-yl]ethanesulfonic acid (HEPES) pH 8, 25 °C, 10 mM ATP, 10 mM UTP, 10 mM GlcA, 20 mM MgCl_2_, 59 µg/mL AtGlcAK, 54 µg/mL AtUSP and 692 µg/mL PmPpA using a volume of 300 µL. Nucleotides and nucleotide sugars were detected with multiplexed capillary electrophoresis (MP-CE).

AtGlcAK, AtUSP, and PmPpA were combined for the one-pot synthesis of UDP–GlcA (Figure 1). UDP–GlcA synthesis reaches 80% and 84% yield after 0.5 and 1 h, respectively. We significantly improved the space-time-yield (STY) reaching 16.1 mmol L^−1^ h^−1^ for UDP–GlcA when compared to a previous study on the same enzyme cascade reporting 89% yield after 12 h and a STY of 1.5 mmol L^−1^ h^−1^ [24]. Li et al. reached 50% yield after 10 h without PmPpA [23]. PmPpA is essential for reaching high product yields and without PmPpA we observed only 32% after 24 h (Figure S5). We want to point out, that we observed a relatively high UDP concentration (1.5 mM) in the purchased UTP [37].

**Figure 2:**
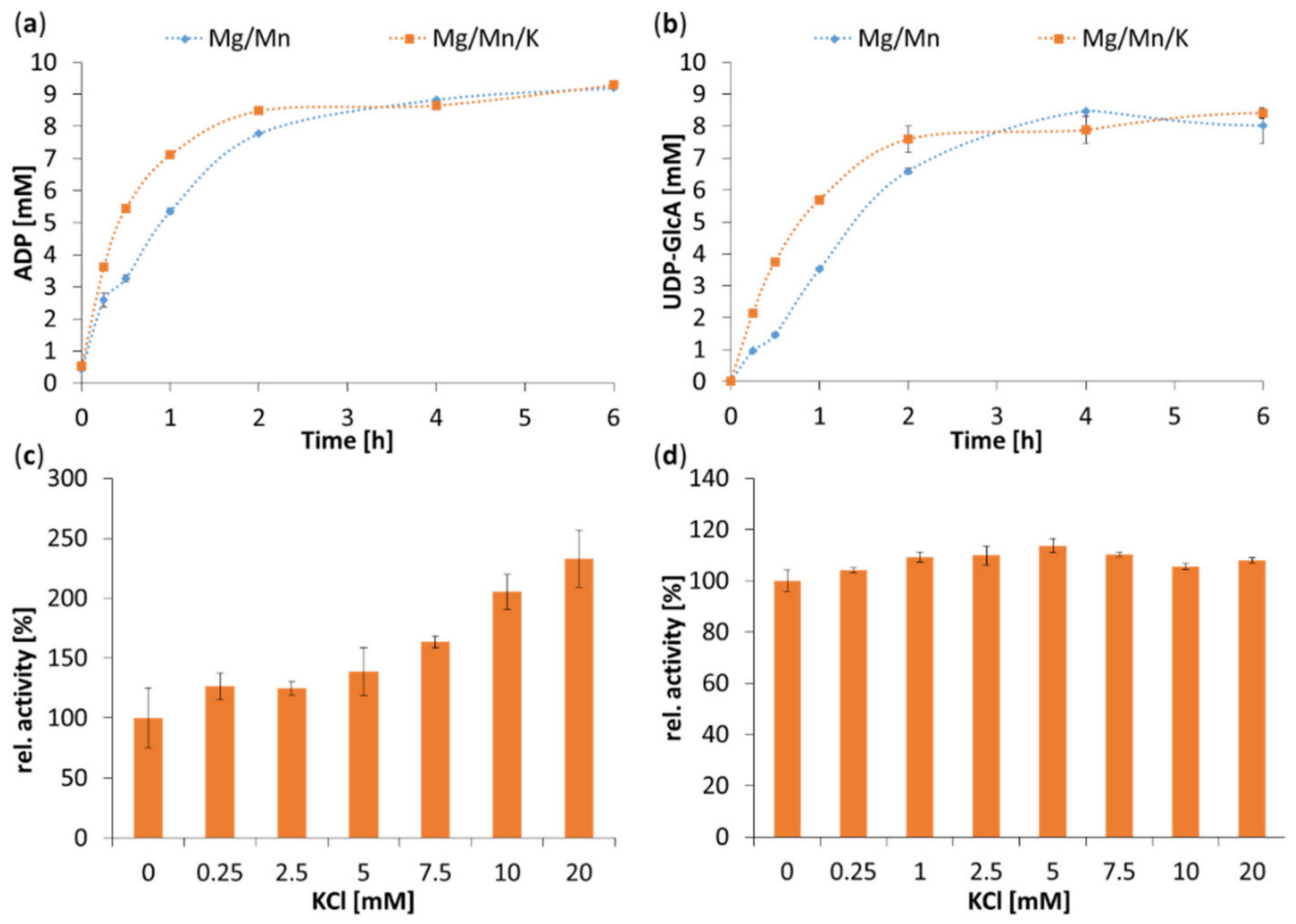
Influence of Mg^2+^, Mn^2+^, and K^+^ on the EM UDP–GlcA. Course of AtGlcAK (**a**) and AtUSP (**b**) reaction within the EM UDP–GlcA with two different cofactor compositions. The reactions included 100 mM HEPES pH 8, 25 °C, 10 mM ATP, 10 mM UTP, 10 mM GlcA, 46 µg/mL AtGlcAK, 92 µg/mL AtUSP, 239 µg/mL PmPpA, and either 10 mM Mg^2+^, and 10 mM Mn^2+^ or 10 mM Mg^2+^, 10 mM Mn^2+^, and 10 mM K^+^. The reaction volume was 300 µL. (**c**): Activity assay for AtGlcAK: 100 mM HEPES pH 8, 25 °C, 5 mM ATP, 5 mM GlcA, 5 mM Mg^2+^, 0–20 mM K^+^, and 62 µg/mL AtGlcAK. 100% activity = 2.34 U/mg. (**d**): Activity assay for AtUSP in the presence of PmPpA: 100 mM HEPES pH 8, 25 °C, 5 mM UTP, 5 mM Glc-1-P, 5 mM Mg^2+^, 0–20 mM K^+^, 58 µg/mL AtUSP, and 18 µg/mL PmPpA using a volume of 300 µL. 100% activity = 121.46 U/mg.

Mn^2+^ is the preferred metal ion for PmHAS, while Mg^2+^ is the preferred metal ion for AtGlcAK and AtUSP [30,36,38]. In our previous work, we found activation of PmHAS by K^+^ in the presence of Mg^2+^ or Mn^2+^ [31]. Therefore, the influence of combining the metal ions Mn^2+^, Mg^2+^, and K^+^ on the enzyme activities and the performance of the EM UDP–GlcA was further investigated (Figure 2).

All combinations of K^+^ with Mg^2+^ and Mn^2+^ gave higher conversion rates of the EM UDP–GlcA within the first hour compared to the corresponding reactions without K^+^ (Figure 2a,b and Figure S6A,B). After 6 h, ~90% yield for ADP and ~85% yield for UDP–GlcA were reached with and without K^+^. When only Mn^2+^ or Mg^2+^ are used as a metal ion, we noticed, that Mn^2+^ has a positive effect on UDP–GlcA production (Figure S6A,B). The AtUSP/PmPpA system seems to prefer Mn^2+^, although AtUSP activity was described to be equal for both metal ions [27]. The kinase AtGlcAK does not prefer one of these cofactors, as described in the literature [30]. Important to note is that after 24 h reactions with Mn^2+^ gave lower yields of UDP–GlcA compared to Mg^2+^ (Figure S6B). Mn^2+^ causes decomposition of UDP–GlcA into the 1,2-cyclic phosphate derivate and uridine monophosphate (UMP) (Figure S6C) [31,39].

To gain more insight, the influence of K^+^ on the enzyme activity of AtGlcAK and AtUSP/PmPpA was investigated (Figure 2c,d). While the activity does not change in the AtUSP/PmPpA system in the presence of K^+^, the AtGlcAK activity is increased 2.3-fold with 20 mM K^+^ compared to the reaction without K^+^. This is a novel feature of glucuronokinases [40,41]. PmHAS also shows higher activity in the presence of K^+^ (Figure S7), confirming our previous study [31]. However, K^+^ has no beneficial effect on the activities of AtUSP, BlNahK, SzGlmU, and PmPpA (Figure S7). A similar, but more intense effect is known for pyruvate kinases, where K^+^ increases the affinity for ADP–Mg^2+^ [42,43]. For human pyridoxal kinase, activity is increased by K^+^ [44,45]. For AtGlcAK we may assume that K^+^ promotes binding of the ATP–Mg^2+^ complex or release of the phosphorylated sugar from the active site. In conclusion, all enzymes of the EM UDP–GlcA are active in the presence of Mn^2+^ and K^+^. Thus, we can apply our previously published optimal conditions for good conversion rates of PmHAS and high molecular weight for HA: these are 15 mM Mg^2+^, 10 mM K^+^, and 1.5 mM Mn^2+^ [31].

### 2.4. One-Pot Synthesis: Combination of the EM UDP–GlcA with the EM HA

**Figure 3:**
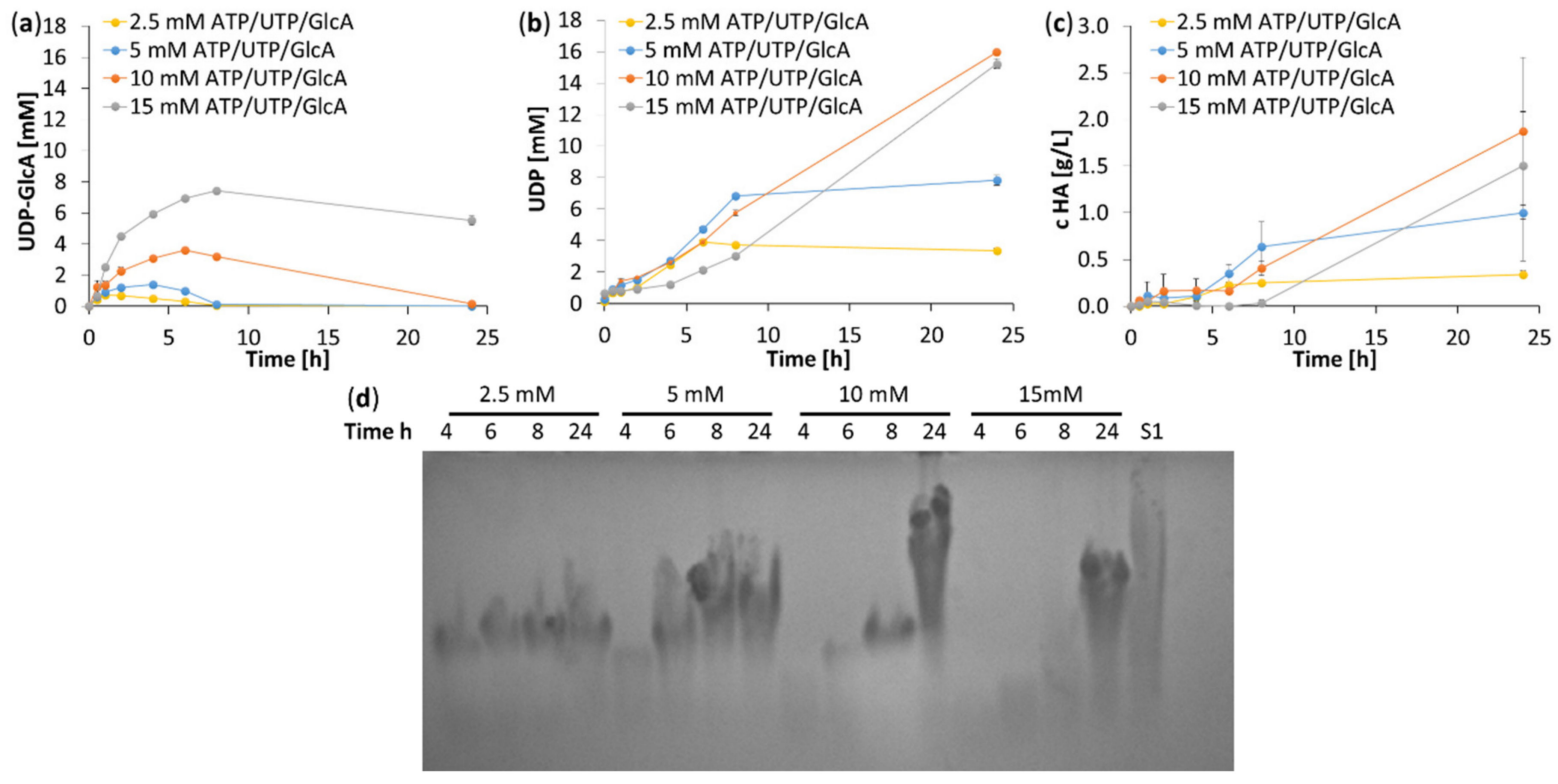
Combination of the EM UDP–GlcA with the EM HA. For the reaction 300 µL were placed in a 96 well plate under the following conditions: 100 mM HEPES pH 8, 25 °C, 15 mM UDP–GlcNAc, X mM ATP/UTP/GlcA, 15 mM Mg^2+^, 10 mM K^+^, 1.5 mM Mn^2+^, 230 µg/mL AtGlcAK, 92 µg/mL AtUSP, 239 µg/mL PmPpA, and 569 µg/mL PmHAS. The course of UDP–GlcA synthesis (**a**) and UDP formation (**b**), as well as the concentration of HA determined with CTM (**c**), and the size of HA determined with agarose gel electrophoresis (**d**), are depicted. S1: high-*M*_w_ HA ≥ 2 MDa, Figure S8 depicts the concentrations of all nucleotides and nucleotide sugars during the time course of the enzymatic reactions.

Kinetic data of PmHAS from our previous study reveal a substrate inhibition by UDP–GlcA (K_Si_ 0.57 mM) and a relatively low affinity for UDP–GlcNAc (K_m_ 23.4 mM) [31]. In general, a high UDP–GlcNAc/UDP–GlcA ratio with a constant supply of UDP–GlcA at low concentrations was favorable for the synthesis of 2 MDa HA. In the present study, we varied substrate concentrations in the EM UDP–GlcA at a constant UDP–GlcNAc concentration (15 mM) to investigate the effect of UDP–GlcA formation on PmHAS activity (formation of UDP) and HA synthesis (Figure 3 and Figure S8).

At low substrate concentrations for the EM UDP–GlcA (2.5 and 5 mM) UDP–GlcA is completely converted after 8 h reaction time (Figure 3a). PmHAS reaches a high activity (0.85 µmol UDP mL^−1^ h^−1^) as calculated from the slope of UDP formation (Figure 3b) and the highest HA size at 5 mM start concentration after 8 h (Figure 3d). At high GlcA concentrations (15 mM) HA formation is limited, the PmHAS activity decreases to 0.38 µmol UDP mL^−1^ h^−1^ and the highest UDP–GlcA concentration of 8 mM after 8 h is reached. At 10 mM substrate concentration, the activity of PmHAS still reaches 0.72 µmol UDP mL^−1^ h^−1^, however, HA size is low after 8 h. In conclusion, these data reflect the activity of PmHAS being high at UDP–GlcA concentrations ≤ 5 mM with immediate HA formation within 8h. At higher UDP–GlcA concentrations, PmHAS activity reaches a limit and shows delayed HA formation. This limit is reached with 10 mM substrate concentrations in the EM UDP–GlcA. Using 10 mM of GlcA, UTP, and ATP, UDP–GlcA is completely converted after 24 h resulting in 1.8 g/L HA and the highest HA size. As a compromise between PmHAS activity, high molecular weight, and concentration after 24 h, 10 mM starting concentrations of substrates in the EM UDP–GlcA was used for the next experiment.

### 2.5. One-Pot Synthesis: Combination of EM UDP–GlcA and EM UDP–GlcNAc with EM HA

**Figure 4:**
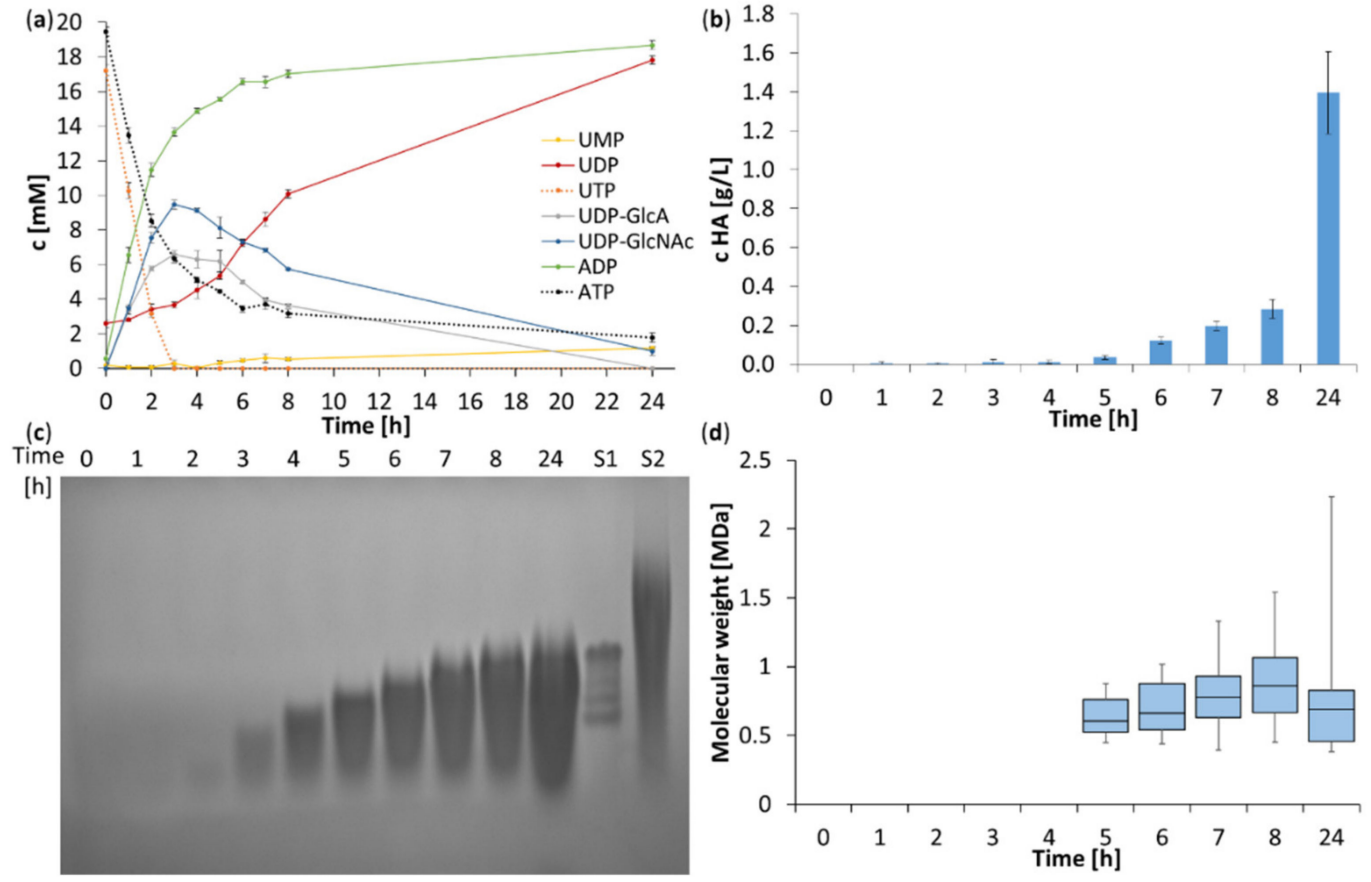
Combination of EM UDP–GlcA and EM UDP–GlcNAc with the EM HA. Conditions: 100 mM HEPES pH 8, 25 °C, 10 mM GlcA, 10 mM GlcNAc, 20 mM ATP, 20 mM UTP, 15 mM Mg^2+^, 10 mM K^+^, 1.5 mM Mn^2+^, 108 µg/mL AtGlcAK, 11 µg/mL AtUSP, 108 µg/mL BlNahK, 109 µg/mL SzGlmU, 46 µg/mL PmPpA, and 1191 µg/mL PmHAS. A volume of 5 mL was used. (**a**): Time course of nucleotide and nucleotide sugar concentrations. (**b**): HA concentration analyzed with CTM. (**c**): HA size analysis by agarose gel electrophoresis with standards S1: Select-HA™ HiLadder (Hyalose LLC) with a composition of defined HA preparations (495, 572, 966, 1090, and 1510 kDa). high-*M*_w_ HA ≥ 2 MDa, S2: high-*M*_w_ HA ≥ 2 MDa. (**d**): Quantitative measurement of the HA-size with SEC-RALS/LALS. Distribution of HA chains’ *M*_w_ is presented as a box plot diagram. The box represents 50% of the HA chains, while each whisker represent 25% of HA chains. The line within the box indicates the median value of HA chains’ *M*_w_.

We combined both nucleotide sugar modules with the HA module in a volume of 5 mL and started the one-pot synthesis with 10 mM GlcA and GlcNAc, respectively (Figure 4). Within 3 h reaction time, UTP is nearly completely converted resulting in ~10 mM UDP–GlcNAc and ~6 mM UDP–GlcA (Figure 4a) starting the production of HA (Figure 4b,c). These concentrations are favorable for an optimal reaction rate of PmHAS (1.26 µmole mL^−1^ h^−1^ UDP) as calculated from the slope of UDP formation (Figure 4a). Interestingly, the size of the HA chain (median values for HA chains’ *M*_w_) gradually increases to *M*_w_ 0.86 MDa within 8 h reaction time (Figure 4d). It is possible to stop the reaction at certain time points to get the preferred HA size. However, the concentration levels of HA are relatively low (Figure 4b). That is also the reason why we could not measure the size of HA before 4 h, although HA is already produced (Figure 4c). Within 8 h, 0.28 g/L HA with an average *M*_w_ of 0.86 MDa and dispersity of 1.11 is reached. After 24 h, the final HA concentration was ~1.4 g/L with an average *M*_w_ of 0.7 MDa and dispersity of 1.16. Interestingly, the maximum HA chain length ranges between 1.54 MDa after 8 h and 2.2 MDa after 24 h. In addition, MP-CE analysis reveals that ~1 mM UMP is formed and ~1 mM UDP–GlcNAc is not utilized after 24 h (Figure 4a). Mn^2+^ degrades UDP–GlcA into a 1,2-cyclic phosphate derivate and UMP. UDP–GlcNAc is stable under these conditions [39]. In summary, ~1 mM UDP–GlcA is not available for the HA synthesis and, because of stoichiometric reasons, 1 mM UDP–GlcNAc are left. Thus 2 mM from 20 mM UTP are not used for the HA production resulting in 90% yield. One way to increase the yields is by varying the Mn^2+^ concentration, although it is already adjusted to the PmHAS activity [31]. The other way is to decrease the pH value because the decomposition is more intense in a basic environment [39]. Compared with the work of Li et al., we reached a higher yield (90% vs. 70%) in shorter reaction time (24 vs. 40 h) [23]. A maximum HA chain length of 0.55 MDa was obtained [23]. These differences are probably due to a different approach to synthesize HA, e.g., equal concentrations of UDP–GlcA and UDP–GlcNAc were applied resulting in a lower activity of PmHAS [31].

### 2.6. Influence of pH on the One-Pot Synthesis of HA

**Figure 5:**
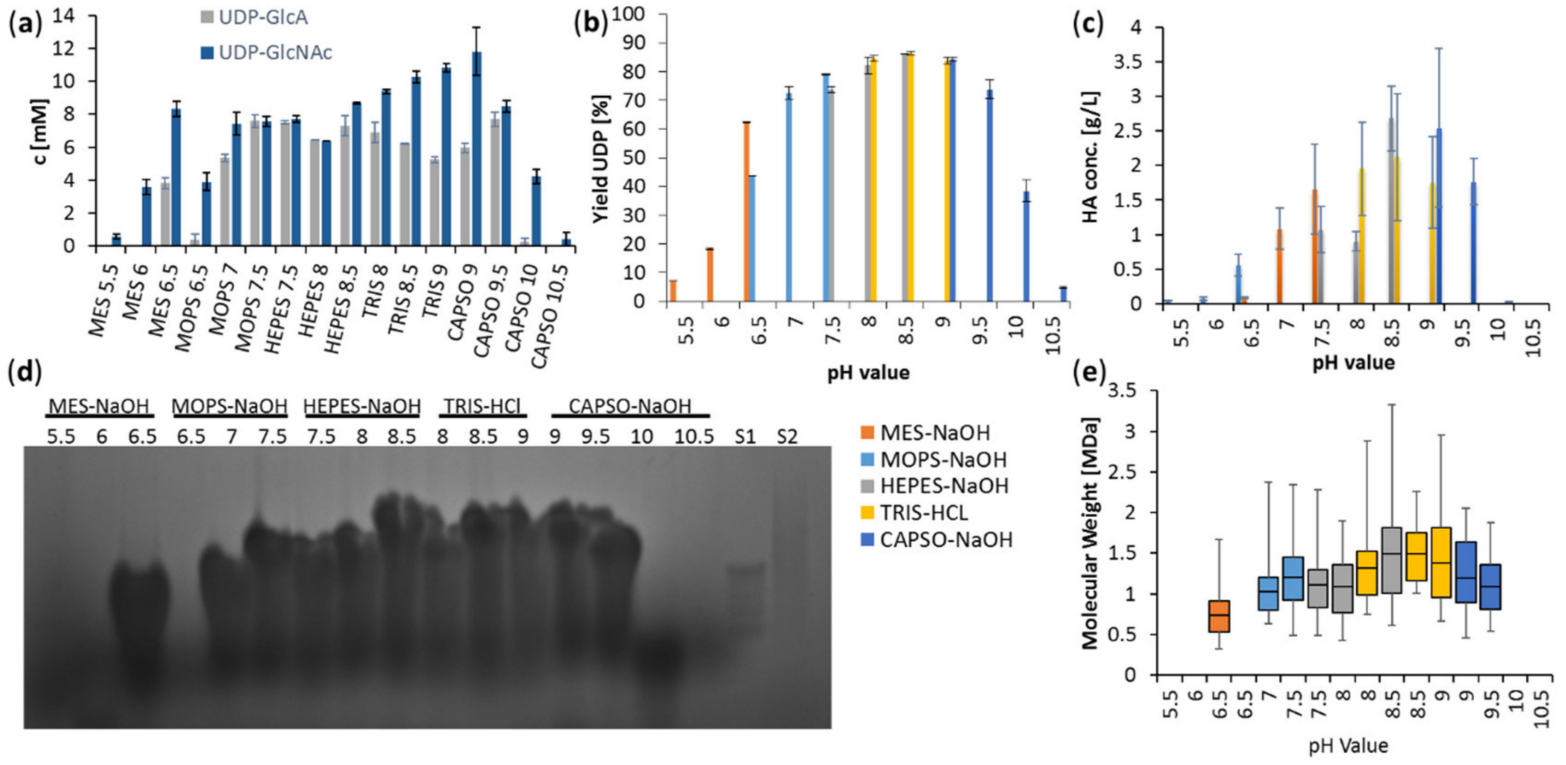
Controlling HA one-pot synthesis with pH value. 100 mM buffer pH X, 25 °C, 10 mM GlcA, 10 mM GlcNAc, 20 mM ATP, 20 mM UTP, 15 mM MgCl_2_, 10 mM KCl, 1.5 mM MnCl_2_, 124 µg/mL AtGlcAK, 117 µg/mL AtUSP, 1202 µg/mL BlNahK, 255 µg/mL SzGlmU, 369 µg/mL PmPpA, 1289 and µg/mL PmHAS. A volume of 5 mL was used. Time course of the reaction of all pH conditions analyzed with MP-CE. All graphs can be found in the supplemental data (Figure S9). (**a**): Concentrations of nucleotide sugars UDP–GlcA and UDP–GlcNAc after 2 h. (**b**): Yield of the reaction after 24 h are calculated by the generated UDP concentration and the UTP starting concentration. (**c**): HA concentration after 24 h measured with CTM. (**d**): Qualitative analysis of the HA-size with agarose gel electrophoresis after 24 h. S1: Select-HA™ HiLadder (Hyalose LLC) with a composition of defined HA preparations (495, 572, 966, 1090, and 1510 kDa), S2: high-*M*_w_ HA ≥ 2 MDa. (**e**): Quantitative measurement of the HA-size with Size-Exclusion Chromatography with Right-Angle/Low-Angle Light Scattering (SEC-RALS/LALS). Distribution of HA chains’ *M*_w_ is presented as a box plot diagram. The box represents 50% of the HA chains, while each whisker represent 25% of HA chains. The line within the box indicates the median value of HA chains’ *M*_w_.

To investigate further the influence of the UDP–sugar ratio on the PmHAS activity in this one-pot synthesis, we varied two system parameters: pH and Mg^2+^. Control of UDP–sugar formation should lead to an optimal HA production with control over HA size. Variation of the pH value shall be a key factor to adjust UDP–sugar ratios since the EM UDP–GlcA and EM UDP–GlcNAc perform differently at specific pH values (Figure 5a and Figure S9). Especially the activity of AtGlcAK is reduced at a pH value higher than 8 (Figure S2D), while the EM UDP–GlcNAc has a broad pH spectrum [31]. After 2 h 10 mM UDP–GlcNAc are produced at pH 8.5 or 9 (Figure 5a), while the UDP–GlcA concentration is lower (5–7 mM). High UDP–GlcNAc/UDP–GlcA ratios lead to higher PmHAS activity as demonstrated for the high UDP yield in Tris-HCl pH 9 (68.8%) after 8 h (Figure S10) reaching final yields of 82% and 86% at pH 8.5 and 9.0, respectively (Figure 5b). These data reflect the relative broad pH optimum of PmHAS as demonstrated in our previous study [31]. As we mentioned earlier, one way to increase the yield could be the decrease of the pH value to reduce the decomposition of UDP–GlcA by Mn^2+^. However, a decrease of the pH value has a greater effect on the functionality of the enzymes (Figure 5b). Final HA concentrations differ for the different buffers at pH values between pH 8 and pH 8.5–9 (Figure 5c). High HA concentrations were obtained at pH 8 with Tris-HCl (1.95 g/L), at pH 8.5 with 2-[4-(2-hydroxyethyl)piperazin-1-yl]ethanesulfonic acid HEPES-NaOH (2.68 g/L), and at pH 9 with 3-(Cyclohexylamino)-1-propanesulfonic acid (CAPSO)-NaOH (2.54 g/L). The size of the HA is also dependent on the pH value (Figure 5d,e). The qualitative analysis showed that the largest HA chains are produced at a pH value of 8.5 and 9 (Figure 5d). This result is confirmed by size exclusion chromatography, showing the highest *M*_w_ with Tris-HCl at pH 8 (1.31 MDa), pH 8.5 (1.50 MDa), and pH 9 (1.38 MDa) (median values for HA chains’ *M*_w_ in Figure 5e and Table S1). The dispersity of these samples in Tris-HCl is between 1.1 and 1.3 (Table S1). We conclude that the best buffer and pH value is HEPES or Tris-HCl at pH 8.0–8.5 for one-pot synthesis of HA in an *M*_w_ range of 1.3–1.5 MDa. Setting this system parameter, the beneficial UDP–GlcA/UDP–GlcNAc ratio in the starting phase of the one-pot synthesis adjusts PmHAS activity for HA polymerization.

### 2.7. Influence of Magnesium on the One-Pot Synthesis of HA

**Figure 6:**
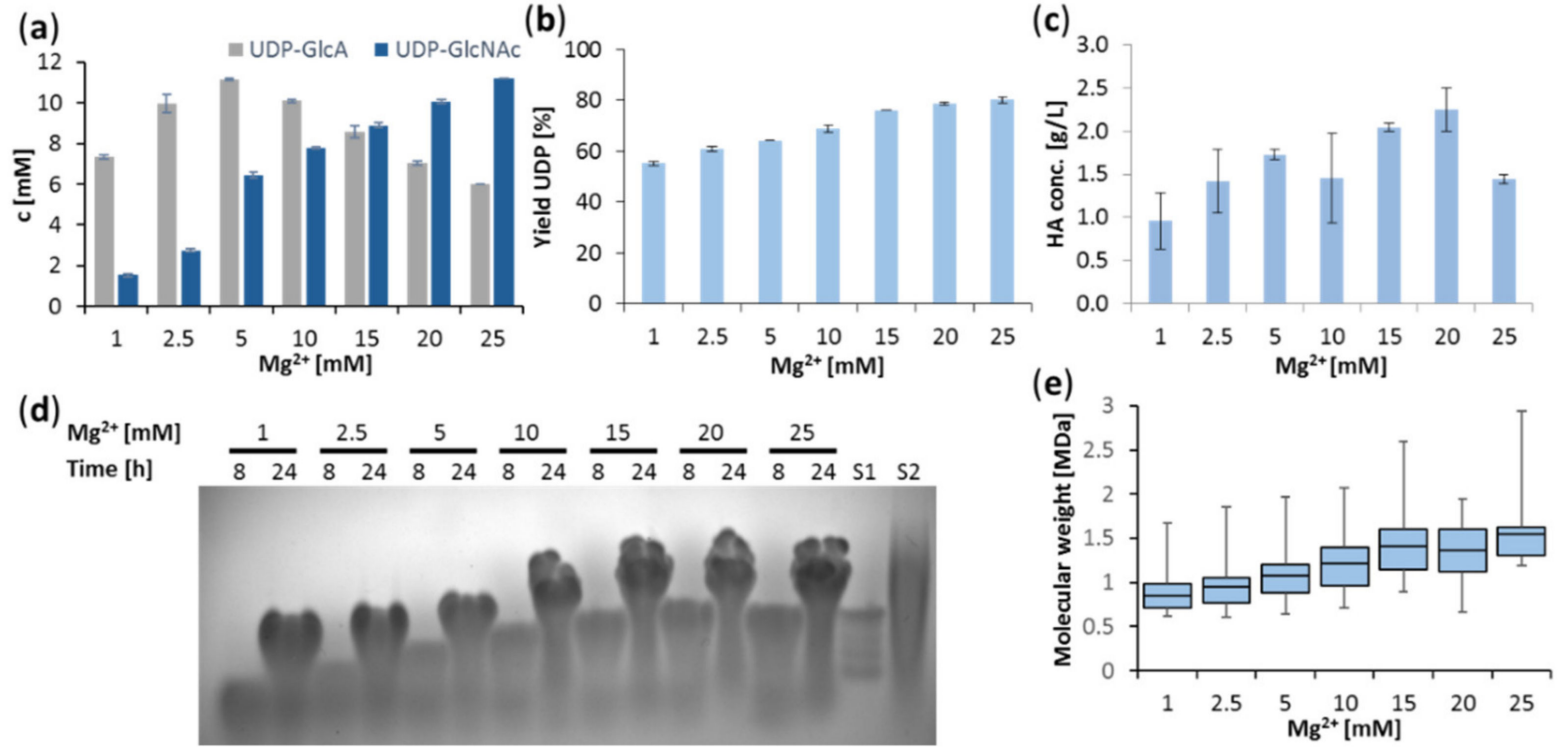
Controlling HA one-pot synthesis with Mg^2+^ concentration. 100 mM HEPES–NaOH pH 8, 25 °C, 10 mM GlcA, 10 mM GlcNAc, 20 mM ATP, 20 mM UTP, X mM MgCl_2_, 10 mM KCl, 1.5 mM MnCl_2_, 1240 µg/mL AtGlcAK, 117 µg/mL AtUSP, 1202 µg/mL BlNahK, 255 µg/mL SzGlmU, 369 µg/mL PmPpA, and 1289 µg/mL PmHAS. A volume of 5 mL was used. (**a)**: Ratio of the intermediate nucleotide sugars UDP–GlcA and UDP–GlcNAc after 2 h. Nucleotides and nucleotide sugars measured with MP-CE. Graphs can be found in the supplemental data (Figure S11). (**b):** Yield of the reaction calculated with the generated UDP concentration and used UTP starting concentration. (**c**): HA concentration after 24 h measured with CTM. (**d**): Qualitative analysis of the HA-size with agarose gel electrophoresis after 8 and 24 h. S1: Select-HA^TM^ HiLadder (Hyalose LLC) with a composition of defined HA preparations (495, 572, 966, 1090, and 1510 kDa), S2: high-*M*_w_ HA ≥ 2 MDa. (**e**): Quantitative measurement of the HA-size with SEC-RALS/LALS. Distribution of HA chains’ *M*_w_ is presented as a box plot diagram. The box represents 50% of the HA chains, while each whisker represent 25% of HA chains. The line within the box indicates the median value of HA chains’ *M*_w_.

In our previous study metal cofactor concentrations were set at 15 mM Mg^2+^, 1.5 mM Mn^2+^ and 10 mM K^+^ for optimal HA synthesis [31]. In the present study using the EM UDP–GlcA, we varied the Mg^2+^ concentration to find the optimal system parameter settings for this one-pot synthesis. (Figure 6 and Figure S11). Most interestingly, a variation of Mg^2+^ concentration has a significant influence on the UDP–GlcNAc/UDP–GlcA ratio (Figure 6a). With 20 mM and 25 mM, a high ratio can be adjusted after 2 h reaction time. At a low Mg^2+^ level (up to 10 mM Mg^2+^), UDP–GlcA is in excess of UDP–GlcNAc. At 15 mM Mg^2+^, both nucleotide sugars are synthesized and converted in equal amounts. These ratios are observed during the entire reaction time (Figure S11) and significantly affect HA synthesis. The ratio of both nucleotide sugars controls the activity of PmHAS as seen in the UDP yield (Figure 6b), HA titer (Figure 6c) as well as HA *M*_w_ (Figure 6d,e, Table S2). While for PmHAS the UDP–GlcNAc concentration should be as high as possible, the optimal UDP–GlcA concentration lays between 3 and 7 mM [31]. The best ratio is reached with 25 mM Mg^2+^ and results in the highest UDP yield (80.2%) (Figure 6b), a high HA concentration (1.49 g/L) (Figure 6c) and the largest HA size (median value for HA chains’ *M*_w_ = 1.55 MDa) (Figure 6e) with an excellent dispersity of 1.049 (Table S2). In contrast, PmHAS activity is lower with an excess of UDP–GlcA over UDP–GlcNAc and results in the formation of lower molecular size HA. Most important, each Mg^2+^ concentration gives a distinct HA *M*_w_ with excellent dispersity and good yield (Figure 6b–e, Table S2). In conclusion, with defined Mg^2+^ concentration in one-pot HA synthesis, we adjust PmHAS activity by the obtained ratios of UDP–GlcNAc and UDP–GlcA and in this way HA size and titer.

Comparing the pH value and the Mg^2+^ concentration as control parameters in the one-pot synthesis of HA, we have different kinds of scenarios. Setting the pH value at pH 8.5–9.0, we adjust a good ratio of the UDP–sugars, but PmHAS does not perform at its pH optimum [31,38]. At 25 mM Mg^2+^ concentration, a beneficial UDP–sugar ratio is reached, and also the activity of PmHAS increases with higher Mg^2+^ concentrations in the presence of K^+^ and Mn^2+^. In both cases, it seems that excess of UDP–GlcNAc over UDP–GlcA gives higher HA yields. Moreover, control over HA polymer size is gained by adjusting the UDP–sugar ratio, preferentially by pH and Mg^2+^ concentration. A similar effect was reported for the microbial HA production with *S. zooepidemicus*, where the addition of GlcNAc or overexpression of genes involved in UDP–GlcNAc synthesis resulted in higher molecular weight of HA, whereas overexpression of UDP–GlcA genes had the opposite effect [46,47,48]. The HAS enzyme in *S. zooepidemicus* is classified as a class 1 type, whereas PmHAS is a class 2 type. [4,18,49]. Both types differ in form, membrane linkage and catalytic reaction [50]. Nevertheless, both enzymes may share biochemical features for HA production and having adapted similarly to their environment [51]. Previous studies showed that PmHAS mode of action could be divided into de novo and polymerization synthesis, where the polymerization step is much faster [13,15]. Studies showed that the GlcA-transferase domain needs at least HA_4_ and the GlcNAc-transferase domain needs at least HA_3_ oligosaccharides for polymerization speed [52]. Interestingly the GlcA-transferase activity is enhanced with longer HA oligosaccharides [22]. HA chain length and polydispersity are greatly dependent on the amount of de novo synthesized chains in the early stages of the reaction. The more HA chains are created, the more the UDP–sugars are distributed among the HA chains. This results in shorter polymers [13,15]. It is possible that a certain UDP–sugar ratio could influence the preference of PmHAS for polymerization or de novo synthesis at the beginning of the reaction, which then would result in different HA sizes after 24 h.

### 2.8. Comparison with Industrial Production Processes

Depending on the demand, the size of the HA chain can be adjusted with the in vitro one-pot synthesis through the UDP–GlcA/UDP–GlcNAc ratio. Another way to regulate the size is, to stop the reaction earlier. The dispersity for all shown reactions is relatively low. With HA extraction from animal tissue, size and dispersity are very hard to control. Both characteristics are hampered trough intrinsically low yields and high dispersity within the tissue and endogenous hyaluronidase activity [53]. Also with bacterial fermentation, low dispersity is still a challenge and is dependent on culture conditions [12,54]. On top of that, extraction from animal tissue and production with pathogenic *Streptococcus* strains bears the risk of biological contamination, which increases the effort and cost of product purification. [3]. The named issues would cease with this in vitro approach.

With the conditions of 15 mM Mg^2+^ and HEPES–NaOH pH 8.5, we could reach an *M*_w_ of 1.49 MDa with a final HA concentration of 2.7 g/L and with 20 mM Mg^2+^ and HEPES–NaOH pH 8 we could reach 1.36 MDa with a final HA concentration of 2.2 g/L. These characteristics can compete with results from bacterial fermentation [55]. However, in our approach, the reaction volume of 5 mL is only lab scale. A scale-up will be necessary for HA production and therefore the preparation of the enzymes will become important [53]. Considering that purification of enzymes is labor and cost-intensive, good long-term storage stability is favorable. Therefore, in the present work the activity of every enzyme was evaluated over four weeks (Figure S7). All enzymes were stored in 100 mM HEPES pH 8 at 4 °C. BlNahK, SzGlmU, and PmHAS are very stable. The AtUSP activity was reduced by ~55% after three weeks. AtGlcAK and PmPpA are most crucial by losing ~70% and ~64% of their activity after one week, respectively. Due to the very high initial activity of PmPpA, this residual activity is still sufficient for HA synthesis. However, the activity loss of AtGlcAK is an issue that will need to be addressed. To enhance the storage stability, immobilization or addition of additives can be used [56]. For example, sucrose enhances the stability of some enzymes significantly [31]. Protein engineering could also help to improve stability or other characteristics as it was recently shown that a PmHAS variant with better activity and chain length specificity was created [57].

## 3. Materials and Methods

Nucleotides and nucleotide sugars are from Carbosynth Limited UK (Compton, UK). All other chemicals are from Carl Roth GmbH (Karlsruhe, Germany), if not further mentioned.

### 3.1. Enzyme Production

#### 3.1.1. Cloning of Recombinant Genes

The synthetic genes of GlcAK from *Arabidopsis thaliana* (Gene ID: 819902), USP from *Arabidopsis thaliana* (Gene ID: 835333) and PpA from *Pasteurella multocida* (Gene ID: 29387852) were modified with restriction recognition sequences (Table 2).

The start codons were removed from the sequence of *glcak* and *ppa* because of the attachment of the *Nde*I sequence, in which is already a start codon. The stop codons were also removed to clone this sequence into the pET-22b(+) vector with C-terminal His_6_-tag. The start codon of *usp* was removed to clone this gene into pET-16b(+) with N-terminal His_10_-tag. All genes were optimized for *Escherichia coli* codon usage and were ordered from GenArt™. The genes were cloned into pET-22b(+) and pET-16b(+), respectively. The cloning was confirmed by sequencing and restriction analysis. The genes for BlNahK from *Bifidobacterium longum* (GenBank accession number: AB303839.1), SzGlmU from *Streptococcus zooepidemicus* (GenBank accession number: AF347022) and HAS^1-703^ from *Pasteurella multocida* (GenBank accession number: AF036004.2) were already cloned into pET-22b(+) vectors in our previous work [31].

#### 3.1.2. Transformation and Cultivation

All constructs pET-22b-*atglcak*, pET-16b-*atusp*, pET-22b-*pmppa,* pET-22b-*blnahk,* pET22b-*szglmu* and pET-22b-*pmhas^1-703^* were transformed into *E. coli* BL21 (DE3) via heat shock. The transformed clones were selected on agar plates with lysogeny broth (LB) medium containing ampicillin (100 µg/mL). Clones were further cultivated in baffled shaking flasks. The pre-culture was grown in 20 mL LB medium with ampicillin (37 °C, 120 rpm). The main culture (1 L) with terrific broth was inoculated with 1% (*v*/*v*) pre-culture (37 °C, 80 rpm). When the OD_600_ of the main culture reached 0.6–0.8, the expression of the proteins was induced by adding 0.1 mM isopropyl β-d-1-thiogalactopyranoside (IPTG, AppliChem GmbH, Darmstadt, Germany) and the temperature was reduced to 25 °C. After 20 h the cells were centrifuged (7000 rpm, 4 °C), the supernatant was discarded and the cells were stored at −20 °C.

#### 3.1.3. Enzyme Purification

Frozen cells (4 g) were suspended with 10 mL binding buffer (20 mM sodium phosphate, 500 mM sodium chloride, 30 mM imidazole, pH 7.4), disrupted with ultrasound and subsequently centrifuged (30 min, 15,000 rpm, 4 °C). The recombinant enzymes containing a His-Tag were purified by immobilized metal ion affinity chromatography (IMAC) using 5 mL HisTrap HP columns from GE Healthcare and the Äktapurifier 100 controlled with the program Unicorn. Target enzymes were eluted using elution buffer (20 mM sodium phosphate, 500 mM sodium chloride, 500 mM imidazole, pH 7.4). The elution buffer was changed by dialysis with 100 mM HEPES pH 8. Protein concentration was measured by the Bradford protein assay. The purification was confirmed by sodium dodecyl sulfate polyacrylamide gel electrophoresis (SDS-PAGE) and Western blot using a His_6_-tag monoclonal antibody, which is conjugated with horseradish peroxidase (Roche Diagnostics GmbH, Mannheim, Germany). The same method for PmHAS and the enzymes of the EM UDP–GlcNAc was used as described in our previous study [31].

### 3.2. Enzyme Assays

#### 3.2.1. Activity Assays

All enzyme activities were tested in a reaction volume of 300 µL in a 96 well plate at 25 °C and pH 8 (100 mM HEPES–NaOH). The composition of each enzyme reaction is listed as follows: AtGlcAK: 5 mM ATP, 5 mM GlcA and 10 mM MgCl_2_. AtUSP: 5 mM UTP, 5 mM Glc-1-P, 10 mM MgCl_2_. Glc-1-P was used instead of GlcA-1-P because of cost reasons. Glc-1-P is hardly available. BlNahK: 5 mM ATP, 5 mM GlcNAc and 10 mM MgCl_2_. SzGlmU: 5 mM UTP, 5 mM GlcNAc-1-P, 10 mM MgCl_2_. PmHAS: 10 mM UDP–GlcA, 10 mM UDP–GlcNAc, 10 mM MnCl_2_. PmPpA: 5 mM PP_i_, 10 mM MgCl_2_. For K^+^ experiments, 10 mM KCl was added. Several samples were taken within 10 min. For PmHAS, several samples were taken within 6 h. Reactions were stopped with a solution consisting of SDS, *para*-aminobenzoic acid (PABA, Sigma Aldrich, Munich, Germany) and *para*-aminophthalic acid (PAPA, Sigma Aldrich) in a ratio of 1:1 resulting in a final concentration of 7 mM SDS, 1 mM PABA and 1 mM PAPA. The reactions for AtGlcAK, AtUSP, BlNahK, SzGlmU, and PmHAS were analyzed with multiplexed capillary electrophoresis and for PmPpA with a phosphate assay kit (MAK308, Sigma Aldrich, Munich, Germany). For the kinases ADP, for AtUSP UDP–GlcA, for SzGlmU UDP–GlcNAc, for PmHAS UDP, and for PmPpA P_i_ as products were monitored. The activity (U = µmol/min) was determined via the slope during the linear phase and the volumetric activity (U/mL) was calculated. With the enzyme concentration from the Bradford assay, the specific activity (U/mg) was calculated.

#### 3.2.2. Kinetic Assays

Kinetics for AtGlcAK, AtUSP, and PmPpA were assayed in a volume of 300 µL in a 96 well plate. The following conditions were used for AtGlcAK: 25 °C, 100 mM HEPES pH 7.5 and 5 mM MgCl_2_. ATP or GlcA, respectively, was set to a constant concentration of 5 mM, while the other one was set in a range from 0.5–30 mM. Kinetics for AtUSP were tested with 5 mM UTP or 5 mM Glc-1-P, while the other substrate was varied between 0.25–25 mM. Conditions were 25 °C, 100 mM HEPES pH 8 and 15 mM MgCl_2_. PmPpA: 25 °C, 100 mM HEPES pH 8. MgCl_2_ concentrations were adjusted to PP_i_ concentrations (1:1). PP_i_ ranged from 1–40 mM. Samples were taken at serval times within 10 min. Reactions were stopped as described for the activity tests. The kinetic parameters were determined with the Michaelis-Menten equation (Equation (1)) or with substrate inhibition (uncompetitive) equation (Equation (2)) using the program SigmaPlot 10.0 (SPSS Software GmbH, Erkrath, Germany).

(1) $$v=V_{\max}\cdot \frac{\left[S\right]}{K_{M}+\left[S\right]}$$

(2) $$v=V_{\max}\cdot \frac{1}{1+\frac{K_{M}}{\left[S\right]}+\frac{\left[S\right]}{K_{\mathrm{iS}}}}$$

[S]: Concentration of the substrate (mM). K_M_: Michaelis constant (mM). *v*: Rate of formation of product (µmol min^−1^ mg^−1^). *V*_max_: Maximal rate of the system (µmol min^−1^ mg^−1^). K_iS_: Dissociation constant of the enzyme-substrate-substrate complex.

#### 3.2.3. One-Pot Syntheses with the EM UDP–GlcA and Whole Cascade

All reactions were tested with 300 µL in a 96 well plate or with 5 mL in a 5.5 mL reaction vessel. All reactions took place at 25 °C. Conditions vary depending on the one-pot synthesis and are directly described in the caption of the figures. Buffers, substrates, cofactors were prepared and a sample of this master mix served as the initial state. Enzymes were added and similar to the activity assays samples were taken at certain time points. Reactions were stopped 1:1 with a solution of SDS, PABA, and PAPA (see 3.2.1 activity assays). Samples were analyzed with MP-CE and if HA was produced, agarose gel electrophoresis, Cetyltrimethylammonium bromide (CTAB) turbidimetric, and SEC-RALS/LALS assays were performed.

### 3.3. Analysis of Enzymatic Syntheses

#### 3.3.1. Multiplexed Capillary Electrophoresis (MP-CE) Analysis

All enzymatic reactions (except for PmPpA) were analyzed by a multiplexed cePRO 9600™ system (Advanced Analytical Technologies, Ames, IA, USA) [35]. The separation of nucleotides and nucleotide sugars for 96 samples was performed in a 96-capillary array, where each capillary had an effective length of 55 cm, a total length of 80 cm and an inner diameter of 50 µm. 10 kV voltage was applied with an electrophoresis buffer (70 mM ammonium acetate, 1 mM EDTA, pH 9.2). Samples were injected by vacuum (−0.7 psi, 10 s). PABA and PAPA were used as an internal standards with a concentration of 1 mM each. The analytes were detected by UV measurement (254 nm) and the resulting data were evaluated with the p*K_a_*-Analyzer software (Advanced Analytical Technologies, Ames, IA, USA). The amount of the analytes were calculated by means of the peak areas.

#### 3.3.2. Phosphate Assay Kit

The enzymatic syntheses with PmPpA were analyzed by a phosphate assay kit (Sigma-Aldrich, MAK308) in accordance with the manufacturer’s instructions. The standard curve ranged from 4–40 µM P_i_ made from a 1 mM P_i_ stock solution. The test sample was diluted to fit in the standard range; 50 µL diluted sample was mixed with 100 µL of the malachite green reagent in a 96 well plate and incubated for 30 min at room temperature. The absorbance was measured at 620 nm.

### 3.4. Analysis of Hyaluronic Acid

#### 3.4.1. Agarose Gel Electrophoresis

The molecular weight of hyaluronic acid was analyzed by agarose gel electrophoresis as described in the literature [58]. Samples and standards were mixed with 4 µL loading buffer (ThermoFisher scientific) and applied on a 0.5% agarose gel. The separation was done with 105 V for 55 min in TAE buffer (40 mM TRIS acetate, 1 mM EDTA, pH 8). In the dark, the gel was washed 30 min with 30% ethanol, dyed for 24 h with Stains-All solution (Sigma-Aldrich, 30% ethanol, 6.25 µg/mL Stains-All), discolored with water and documented at the GelDoc (BioRad, Düsseldorf, Germany). Two markers were used: (a) Select-HA™ HiLadder (Hyalose LLC) with a composition of defined HA preparations (495, 572, 966, 1090 and 1510 kDa), (b) high-*M*_w_ HA ≥ 2 MDa (Contipro Biotech, Dolní Dobrouč, Czech Republic).

#### 3.4.2. CTAB Turbidimetric Method (CTM)

CTM is a method to measure the concentration of HA, which is based on the formation of turbidity between HA and cetyltrimethylammonium bromide (CTAB) [59,60,61]. 25 µL of acetate buffer (200 mM sodium acetate, 150 mM sodium chloride, pH 6) were mixed with 25 µL sample (1:20 dilution). CTAB solution of 100 µL (25 g/L CTAB in 2% *w*/*v* sodium hydroxide) was added, incubated for 5 min at room temperature and the readings were taken at 400 nm. From all absorption values, the associated *t* = 0 min value was subtracted and the HA concentration was calculated with linear regression. The standard curve was provided by dilutions of high-*M*_w_ HA (Contipro Biotech, *M*_w_ ≥ 2 MDa) ranging from 6.25–200 µg/mL.

#### 3.4.3. Size-Exclusion Chromatography with Right-Angle/Low-Angle Light Scattering (SEC-RALS/LALS)

SEC-RALS/LALS (Viscotek TDA 305, Malvern Instruments, Kassel, Germany) was used to determine the molecular weight distribution (*M*_w_ and *M_n_*) and relative molecular mass dispersity within the HA samples, by using two linearly connected columns (A6000M, A7000, Malvern Instruments) for separation. Samples were diluted with 10 mM PBS (pH 7.4) up to a volume of 180 µL. 80 µL of this dilution was injected and were eluted with PBS at a flow rate of 0.35 mL/min and 35 °C. HA concentrations were determined by RI-detector. PEO standard (0.5 mg/mL in PBS, Malvern Instruments) was used for the calibration. The OmniSEC software employs triple detection (RI, RALS/LALS) and allows for a one-point calibration. We analyzed each sample twice and results are the mean value of two runs. The dispersity was calculated with the quotient of *M*_w_ and *M_n_* (Equation (5)).

(3) $$Mn=\;\frac{\sum ni\cdot Mi}{\sum ni}$$

(4) $$Mw\;=\;\frac{\sum mi\cdot Mi}{\sum mi}$$

(5) $$DI=\;\frac{Mw}{Mn}$$

*M_i_*: Molar mass of chains with i units. *n_i_*: Number of chains with i units. *m_i_*: Total mass of chains with i units. DI: Dispersity index

## 4. Conclusions

In this study, we aimed to understand and control the key factors for complex one-pot synthesis of HA. The one-pot synthesis contains six different enzymes and the HA chain is built up from the cheap monosaccharides GlcA and GlcNAc. As an advanced development, we present an optimized one-pot synthesis including the insights we got in our previous study [31]. For the one-pot synthesis, we used a specific metal ion composition of Mg^2+^, Mn^2+^, and K^+^, adjusted for the combination of PmHAS and the EM UDP–GlcNAc. Surprisingly, we found out that K^+^ enhances also the activity of AtGlcAK, a novel feature for this plant glucuronokinase. The pH value and Mg^2+^ concentration are key factors to control HA synthesis. Depending on these key factors, we could indeed tailor HA size, yield, and dispersity. We obtained high molecular weight HA (1.54 MDa) with low dispersity (1.05) at high (25 mM) Mg^2+^ concentrations in HEPES–NaOH buffer (pH 8). The yield was 80.2% with a final HA concentration of 1.4 g/L. High HA concentration (2.7 g L^−1^) is reached with 15 mM Mg^2+^ in HEPES–NaOH (pH 8.5) with a yield of 86.3%, an average size of 1.49 MDa and a dispersity of 1.2. Future development should include scale-up, where immobilization of the enzymes may become important. In addition, the development of a regeneration system for the nucleotides ATP and UTP is demanding to reduce costs [62,63]. In conclusion, we demonstrated that manufacturing of high *M*_w_ and low dispersity HA product can be tailored with an in vitro one-pot synthesis.

## Figures and Tables

**Table 1:** Kinetic data of enzymes in the EM UDP–GlcA.

Kinetic Constants ^1^	AtGlcAK	AtUSP	PmPpA
*V*_max_ (U/mg)	GlcA: 7.70; ATP_app_^2^: 35.83	Glc-1-P: 129.66 UTP: 156.33	PP_i, app_^2^: 668000
*K*_m_ (mM)	GlcA: 0.62 ATP_app_^2^: 8.56	Glc-1-P: 0.58 UTP: 0.44	PP_i, app_^2^: 3552.58
*K*_iS_ (mM)	GlcA: - ATP: 2.87	-	PP_i, app_^2^: 0.0086

^1^ Kinetic constants were calculated with equation 1 and 2.^2^ Apparent kinetic constants.

**Table 2:** Modification of recombinant genes.

Gene	N-Terminal	C-Terminal
*glcak*	*Nde*I GGAATTCCATATGGAATTCC	*Xho*I CCGCTCGAGCGG
*usp*	*Xho*I CCGCTCGAGCGG	*BamH*I CGGGATCCCG
*ppa*	*Nde*I GGAATTCCATATGGAATTCC	*Xho*I CCGCTCGAGCGG

## Supplementary Materials

Supplementary materials can be found at https://www.mdpi.com/1422-0067/20/22/5664/s1.

## Abbreviations

ADP	Adenosine diphosphate
AMP	Adenosine monophosphate
AtGlcAK	Glucuronic acid kinase from *Arabidopsis thaliana*
ATP	Adenosine triphosphate
AtUSP	UDP–sugar pyrophosphorylase from *Arabidopsis thaliana*
BlNahK	GlcNAc-1-phosphate kinase from *Bifidobacterium longum*
CAPSO	3-(Cyclohexylamino)-1-propanesulfonic acid
CjGlmU	UDP–GlcNAc pyrophosphorylase from *Campylobacter jejuni*
CTAB	Cetyltrimethylammonium bromide
CTM	CTAB turbidimetric method
EM	Enzyme module
GlcA	Glucuronic acid
GlcNAc	*N*-acetylglucosamine
HA	Hyaluronic acid
HCl	Hydrogen chloride
HEPES	2-[4-(2-Hydroxyethyl)piperazin-1-yl]ethanesulfonic acid
IMAC	Immobilized metal affinity chromatography
IPTG	Isopropyl β-d-1-thiogalactopyranoside
K^+^	Potassium cation
MES	2-Morpholin-4-ylethanesulfonic acid
Mg^2+^	Magnesium cation
Mn^2+^	Manganese cation
MOPS	3-Morpholinopropane-1-sulfonic acid
MP-CE	Multiplexed capillary electrophoresis
*M*_W_	Molecular weight
NaOH	Sodium hydroxide
PABA	*para*-Aminobenzoic acid
PAPA	*para*-Aminophthalic acid
PBS	Phosphate-buffered saline
P_i_	Inorganic phosphate
PmHAS	Hyaluronan synthase from *Pasteurella multocida*
PmPpA	Pyrophosphatase from *Pasteurella multocida*
PP_i_	Inorganic Pyrophosphate
SDS-PAGE	Sodium dodecyl sulfate polyacrylamide gel electrophoresis
SEC-RALS/LALS	Size-Exclusion Chromatography with Right-Angle/Low-Angle Light Scattering
SzGlmU	UDP–GlcNAc pyrophosphorylase from *Streptococcus zooepidemicus*
TRIS	2-Amino-2-(hydroxymethyl)propane-1,3-diol
UDP	Uridine diphosphate
UMP	Uridine monophosphate
UTP	Uridine triphosphate
UV	Ultraviolet
