# Supplementary material for: Key Factors for a One-Pot Enzyme Cascade Synthesis of High Molecular Weight Hyaluronic Acid
Source: Int J Mol Sci. 2019 Nov 12;20(22):5664. doi: 10.3390/ijms20225664 (PMC6888640; doi:10.3390/ijms20225664)
Supplement: Supplementary file 1 [file ijms-20-05664-s001.pdf]

Article

Key Factors for a One-Pot Enzyme Cascade Synthesis of High Molecular Weight Hyaluronic Acid

Johannes Gottschalk [1], Henning Zaun [2], Anna Eisele [1], Jürgen Kuballa [2], Lothar Elling [1]*

[1] Laboratory for Biomaterials, Institute of Biotechnology and Helmholtz-Institute for Biomedical Engineering, RWTH Aachen University, Pauwelsstraße 20, 52074 Aachen (Germany), E-mail: l.elling@biotec.rwth-aachen.de

[2] Research and Development Department, GALAB Laboratories GmbH, Am Schleusengraben 7, 21029 Hamburg (Germany), E-mail: juergen.kuballa@galab.de

* Prof. L. Elling: l.elling@biotec.rwth-aachen.de

1 Enzyme Production

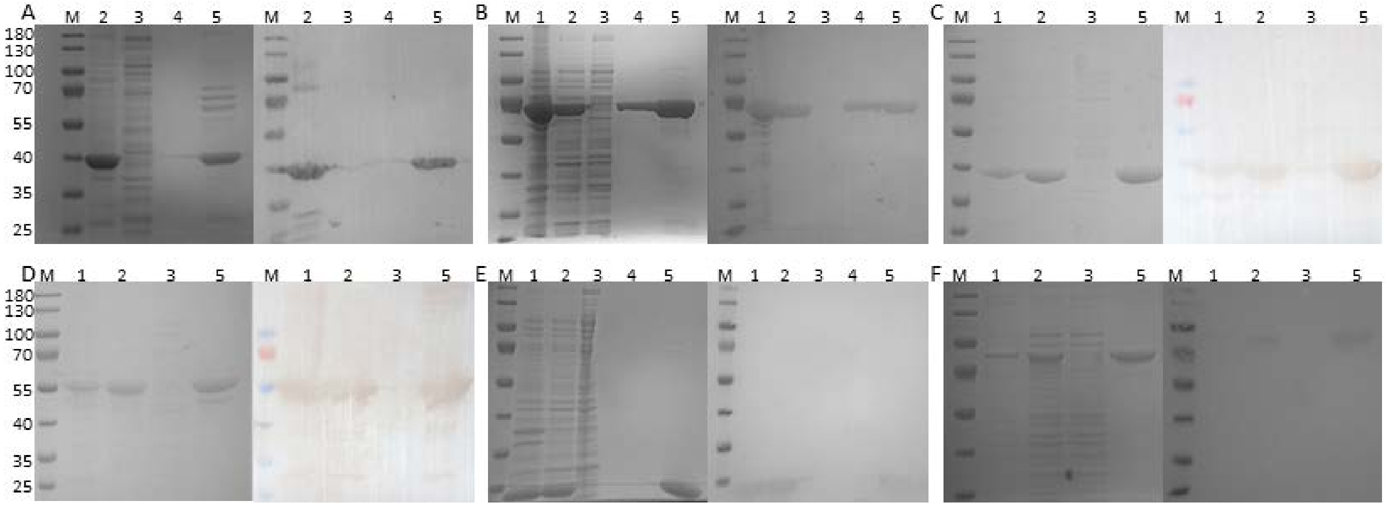

Figure S1. SDS-PAGE and Western blot. M: PageRuler™ 180 kDa, 1: solved cell mass, 2: crude extract, 3: flow-through, 4: permeate after filtration 5: retentate after filtration. A: AtGlcAK-His$_6$ calculated mass: 41.5 kDa, B: His$_{10}$-AtUSP calculated mass: 70.8 kDa, C: BlNahK-His$_6$ calculated mass: 41.3 kDa, D: GlmU-His$_6$ calculated mass: 50.6 kDa, E: PmPpA-His$_6$ calculated mass: 20.8, F: PmHAS$^{1\text{-}703}$-His$_6$ calculated mass: 82.1 kDa.

2 Characterization of the UDP-GlcA Module

2.1 Characterization of AtGlcAK

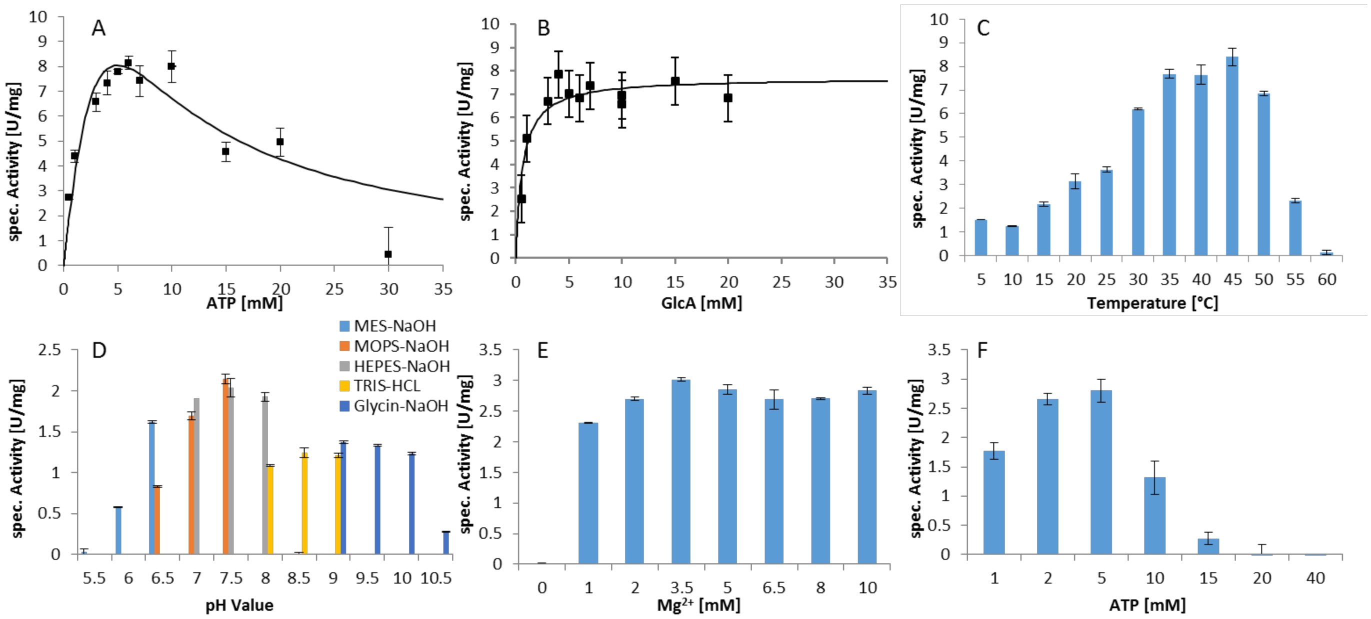

Figure S2. Characterization of AtGlcAK including kinetics, temperature, pH and Mg^{2+} dependency. Analyzed with MP-CE. A: 5 mM GlcA, X mM ATP, 5 mM Mg^{2+}, 12.42 µg/mL AtGlcAK, 100 mM HEPES pH 7.5, 25 °C. V_{max} = 35.83 U/mg, K_m = 8.56 mM, K_{iS} (ATP) = 2.87 mM. R^2 = 0.80. **B**: 5 mM ATP, X mM GlcA, 5 mM Mg^{2+}, 12.42 µg/mL AtGlcAK, 100 mM HEPES pH 7.5, 25 °C. V_{max} = 7.70 U/mg, K_m = 0.62 mM, R^2 = 0.84. **C**: 5 mM GlcA, 5 mM ATP, 5 mM Mg^{2+}, 20.73 µg/mL AtGlcAK, 100 mM HEPES pH 7.5, X °C. **D**: 5 mM GlcA, 5 mM ATP, 5 mM Mg^{2+}, 20.73 µg/mL AtGlcAK, 100 mM buffer pH X, 25 °C. **E**: 5 mM GlcA, 5 mM ATP, X mM Mg^{2+}, 20.73 µg/mL AtGlcAK, 100 mM HEPES pH 7.5, 25 °C. **F**: 5 mM GlcA, X mM ATP, X Mg^{2+}, 12.42 µg/mL AtGlcAK, 100 mM HEPES pH 7.5, 25 °C.

2.2 Characterization of AtUSP

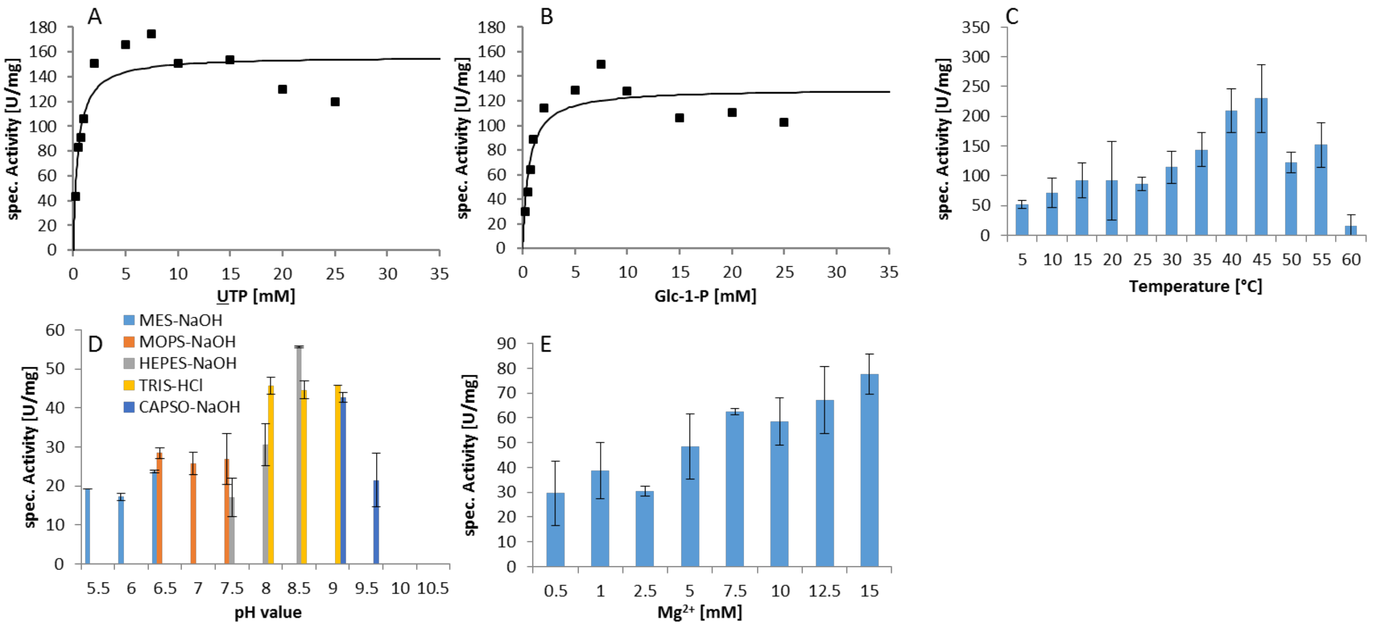

Figure S3. Characterization of AtUSP including kinetics, temperature, pH and Mg^{2+} dependency. Analyzed with MP-CE. A: 5 mM Glc-1-P, X mM UTP, 15 mM Mg^{2+}, 1.31 µg/mL AtUSP, 100 mM HEPES pH 8, 25 °C. V_{max} = 156.33 U/mg, K_m = 0,44 mM, R^2 = 0.78. **B**: X Glc-1-P, 5 mM UTP, 15 mM Mg^{2+}, 1.31 µg/mL, 100 mM HEPES pH 8, 25 °C. V_{max} = 129.66 U/mg, K_m = 0.58 mM, R^2 = 0.79. **C**: 5 mM Glc-1-P, 5 mM UTP, 10 mM Mg^{2+}, 0.44 µg/mL AtUSP, 100 mM HEPES pH 8, X °C. **D**: 5 mM Glc-1-P, 5 mM UTP, 10 mM Mg^{2+}, 1.36 µg/mL AtUSP, 100 mM buffer pH X, 25 °C. **E**: 5 mM Glc-1-P, 5 mM UTP, X mM Mg^{2+}, 0.87 µg/mL AtUSP, 100 mM HEPES pH 8, 25 °C.

2.3 Characterization of PmPpA

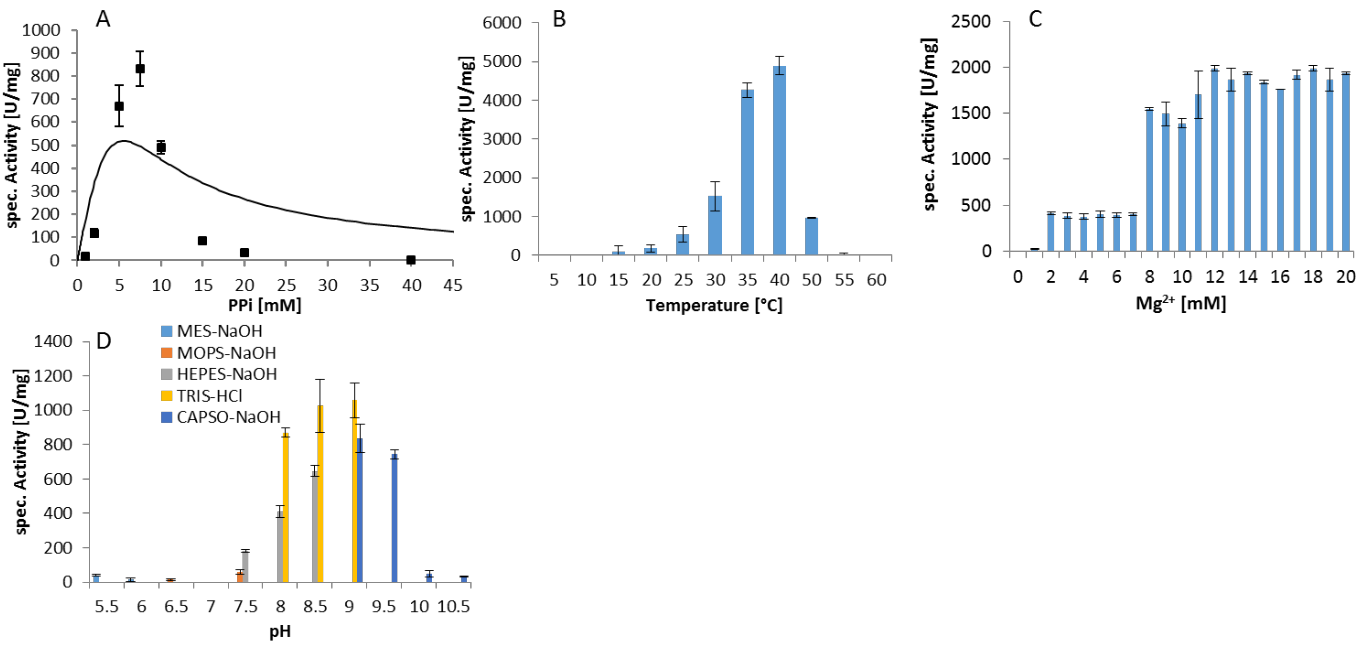

Figure S4. Characterization of PmPpA including kinetics, temperature, pH and Mg^{2+} dependency. Analyzed with phosphate assay kit. A: X mM PPi, X mM Mg^{2+}, 14.12 µg/mL PmPpA, 100 mM HEPES pH 8, 25 °C. V_{max} = 668000 U/mg, K_M = 3552.58 mM, K_{iS} = 0.0086 mM, R^2 = 0.55. **B**: 5 mM PPi, 5 mM Mg^{2+}, 14.12 µg/mL PmPpA, 100 mM HEPES-NaOH pH 8, X °C. **C**: 5 mM PPi, X mM Mg^{2+}, 14.12 µg/mL PmPpA, 100 mM HEPES pH 8, 25 °C. **D**: 5 mM PPi, 10 mM Mg^{2+}, 1.80 µg/mL PmPpA, 100 mM buffer pH X, 25 °C.

3 UDP-GlcA Module One-Pot Synthesis

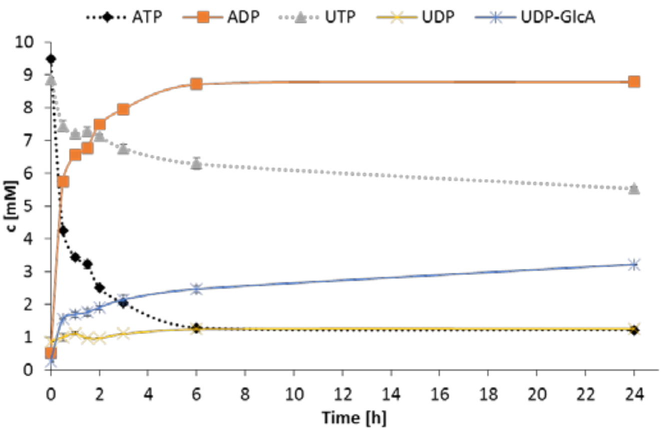

Figure S5. Synthesis of UDP-GlcA with AtGlcAK, and AtUSP. One-pot synthesis was performed under the following conditions: 100 mM HEPES pH 8, 25 °C, 10 mM ATP, 10 mM UTP, 10 mM GlcA, 20 mM $MgCl_2$, 117.76 µg/mL AtGlcAK and 107.47 µg/mL AtUSP using a volume of 300 µL. Nucleotides and nucleotide sugars were detected with MP-CE.

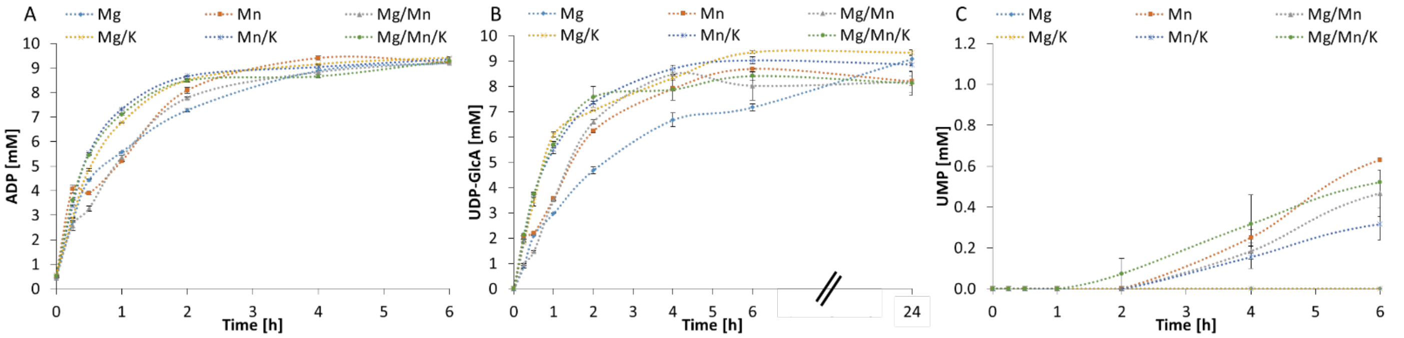

Figure S6. Influence of Mn^{2+} and K^{+} on the EM UDP-GlcA. Course of ADP (**A**), UDP-GlcA (**B**), and UMP (**C**); UDP-GlcA module with different cofactor compositions: The reactions contained 100 mM HEPES-NaOH pH 8, 25 °C, 10 mM ATP, 10 mM UTP, 10 mM GlcA, different combinations of 10 mM Mg^{2+}, 10 mM Mn^{2+}, and 10 mM K^{+}, 45.61 µg/mL AtGlcAK, 92.49 µg/mL AtUSP and 238.86 µg/mL PmPpA. A volume of 300 µL was used. Nucleotides and nucleotide sugars were measured with MP-CE.

4 Long Term Stability and Influence of K^{+}

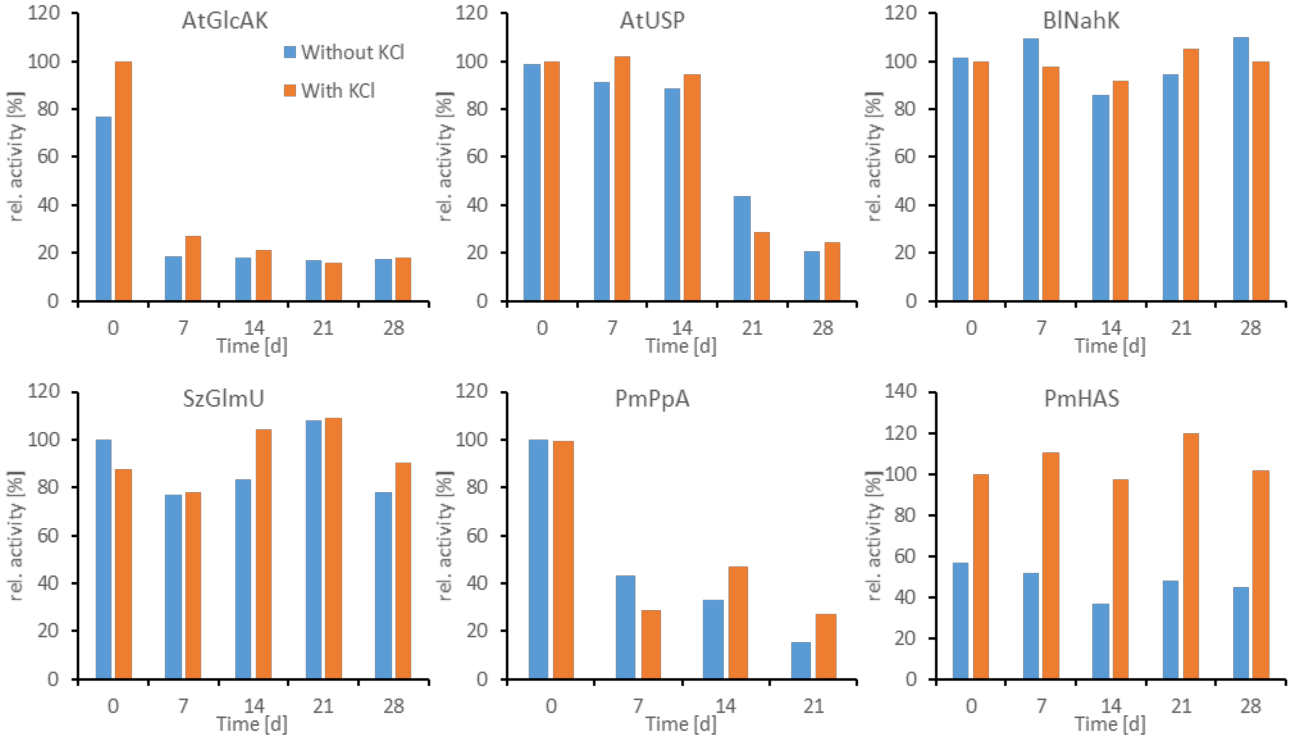

Figure S7. Long-term stability of all six enzymes and their dependence on K^{+}. The activity assays were performed in 300 µL at 25 °C with 100 mM HEPES pH 8. The substrates and cofactors vary depending on the enzyme: **AtGlcAK**: 5 mM ATP, 5 mM GlcA and 10 mM Mg^{2+}, 61.63 µg/mL AtGlcAK, 100 % = 0.68 U/mg. **AtUSP**: 5 mM UTP, 5 mM Glc-1-P, 10 mM Mg^{2+}, 13.07 µg/mL AtUSP, 100 % = 11.42 U/mg. **BlNahK**: 5 mM ATP, 5 mM GlcNAc and 10 mM Mg^{2+}, 121.29 µg/mL BlNahK, 100 % = 0,93 U/mg. **SzGlmU**: 5 mM UTP, 5 mM GlcNAc-1-P, 10 mM Mg^{2+}, 125.52 µg/mL SzGlmU, 100 % = 0.55 U/mg. **PmPpA**: 5 mM PPi, 10 mM Mg^{2+}, 0.31µg/mL PmPpA, 100 % = 1195.00 U/mg. **PmHAS**: 10 mM UDP-GlcA, 10 mM UDP-GlcNAc, 10 mM Mn^{2+},158.17 µg/mL PmHAS, 100 % = 0.087 U/mg. For potassium experiments 10 mM K^{+} were added. Samples were taken during a period of 10 min or 6 h for PmHAS, respectively. The reaction of PmPpA was analyzed with a phosphate assay kit and all other reactions with multiplexed capillary electrophoresis.

5 Combination of the UDP-GlcA Module with the HA Module

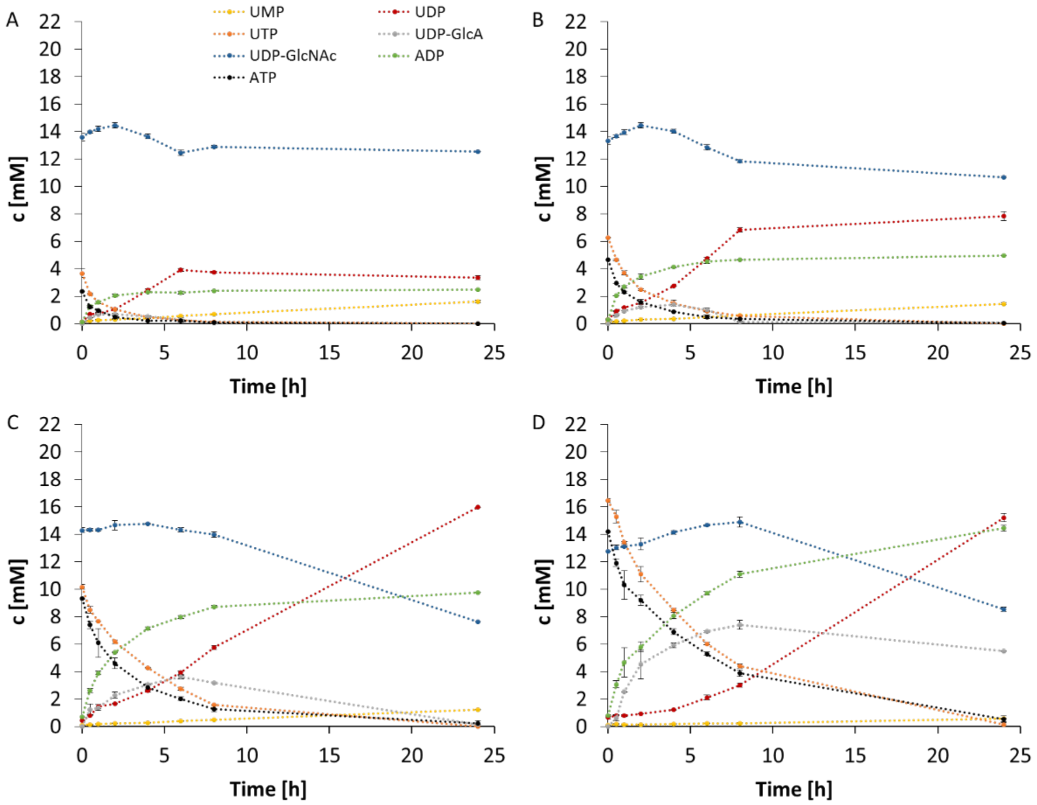

Figure S8. Combination of the UDP-GlcA module with the HA module. For the reaction 300 µL were placed in a 96 well plate under the following conditions: 100 mM HEPES pH 8, 25 °C, 15 mM UDP-GlcNAc, 2.5 (**A**), 5 (**B**), 10 (**C**), and 15 mM (**D**) ATP/UTP/GlcA, 15 mM Mg^{2+}, 10 mM K^{+}, 1.5 mM Mn^{2+}, 22.80 µg/mL AtGlcAK, 92.49 µg/mL AtUSP, 238.86 µg/mL PmPpA, and 568.50 µg/mL PmHAS. Nucleotides and nucleotide sugars were analyzed with MP-CE.

6 Influence of pH on the One-Pot Synthesis

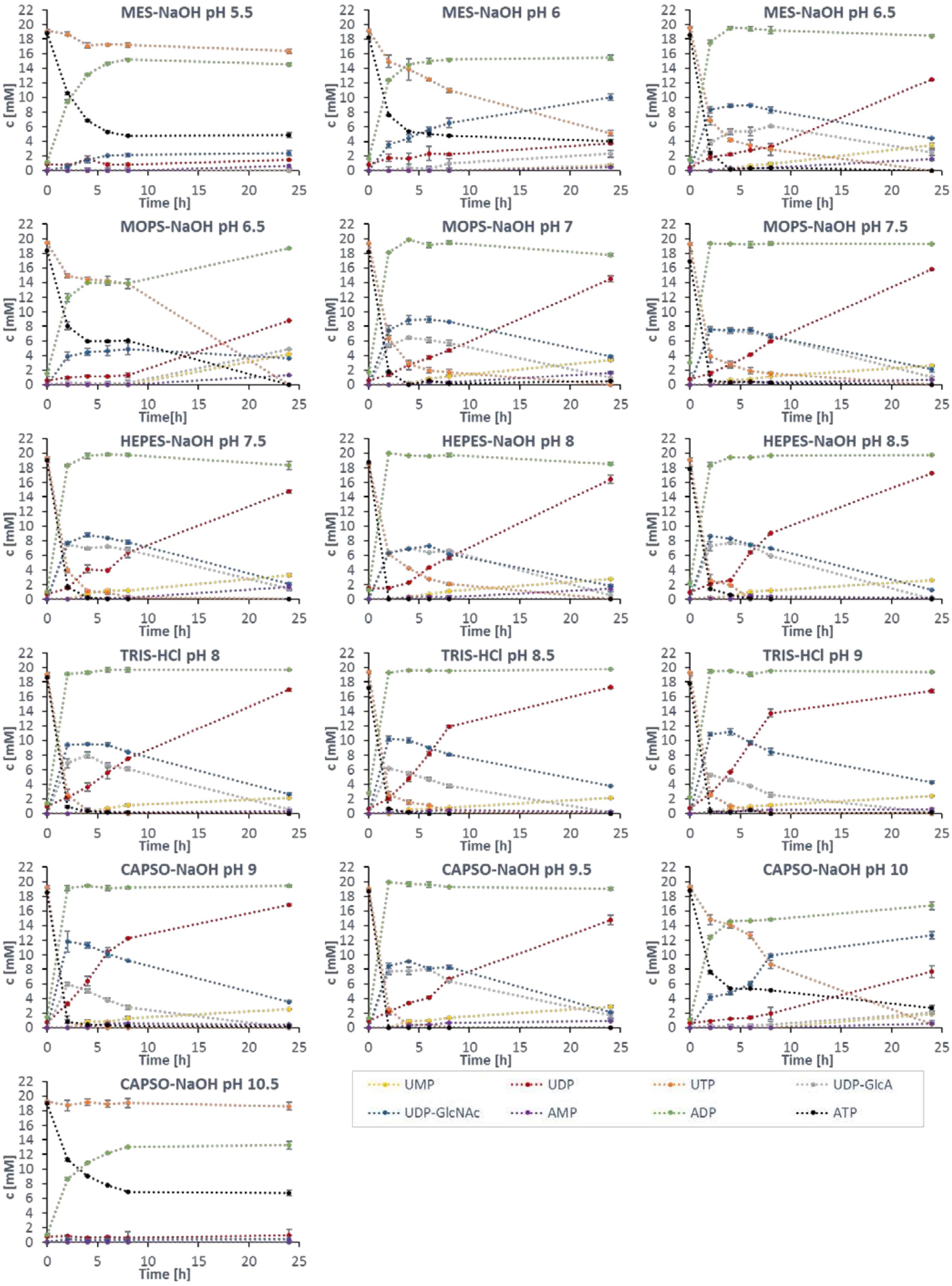

Figure S9. Controlling HA one-pot synthesis with pH value. 100 mM buffer pH X, 25 °C, 10 mM GlcA, 10 mM GlcNAc, 20 mM ATP, 20 mM UTP, 25 mM $MgCl_2$, 10 mM KCl, 1.5 mM $MnCl_2$ 123.50 µg/mL AtGlcAK, 116.71 µg/mL AtUSP, 1202.42 µg/mL BlNahK, 254.63 µg/mL GlmU, 369.44 µg/mL PmPpA, 1289.14 µg/mL PmHAS. A volume of 5 mL was used. The reaction of specific pH values was analysed with MP-CE over time.

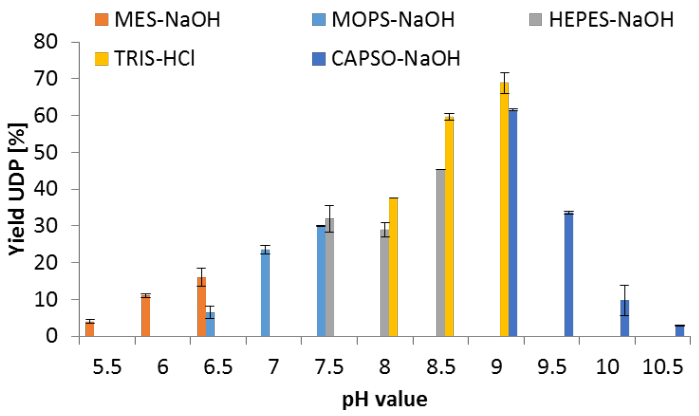

Figure S10. Controlling HA one-pot synthesis with pH value. 100 mM buffer pH X, 25 °C, 10 mM GlcA, 10 mM GlcNAc, 20 mM ATP, 20 mM UTP, 15 mM $MgCl_2$, 10 mM KCl, 1.5 mM $MnCl_2$, 123.50 µg/mL AtGlcAK, 116.71 µg/mL AtUSP, 1202.42 µg/mL BlNahK, 254.63 µg/mL SzGlmU, 369.44 µg/mL PmPpA, 1289.14 µg/mL PmHAS. A volume of 5 mL was used. Yield of the reaction after 8 h calculated with the generated UDP concentration and used UTP start concentration.

Table S1. Controlling HA one-pot synthesis by pH. HA size measurement with SEC-RALS/LALS. Samples were taken after 24 h.

Buffer - pH	Mw	Mn	Q	Max	Max 75 %	Min 25 %	Min
MES-NaOH 5.5	N.D.	N.D.	N.D.	N.D.	N.D.	N.D.	N.D.
MES-NaOH 6.0	N.D.	N.D.	N.D.	N.D.	N.D.	N.D.	N.D.
MES-NaOH 6.5	0.735	0.612	1.201	1.674	0.91	0.53	0.325
MOPS-NaOH 6.5	N.D.	N.D.	N.D.	N.D.	N.D.	N.D.	N.D.
MOPS-NaOH 7.0	1.026	0.936	1.096	2.378	1.198	0.802	0.631
MOPS-NaOH 7.5	1.205	1.052	1.145	2.341	1.45	0.919	0.493
HEPES-NaOH 7.5	1.109	0.96	1.155	2.285	1.3	0.832	0.489
HEPES-NaOH 8.0	1.09	0.887	1.229	1.902	1.36	0.77	0.423
HEPES-NaOH 8.5	1.489	1.22	1.220	3.322	1.81	1.011	0.61
TRIS-HCl 8.0	1.313	1.172	1.120	2.88	1.52	0.986	0.745
TRIS-HCl 8.5	1.497	1.385	1.081	2.256	1.75	1.157	1.001
TRIS-HCl 9.0	1.375	1.08	1.273	2.952	1.81	0.958	0.661
CAPSO-NaOH 9.0	1.194	0.973	1.227	2.051	1.634	0.89	0.459
CAPSO-NaOH 9.5	1.086	0.957	1.135	1.876	1.362	0.813	0.54
CAPSO-NaOH 10	N.D.	N.D.	N.D.	N.D.	N.D.	N.D.	N.D.
CAPSO-NaOH 10.5	N.D.	N.D.	N.D.	N.D.	N.D.	N.D.	N.D.

7 Influence of Mg^{2+} on the One-Pot Synthesis

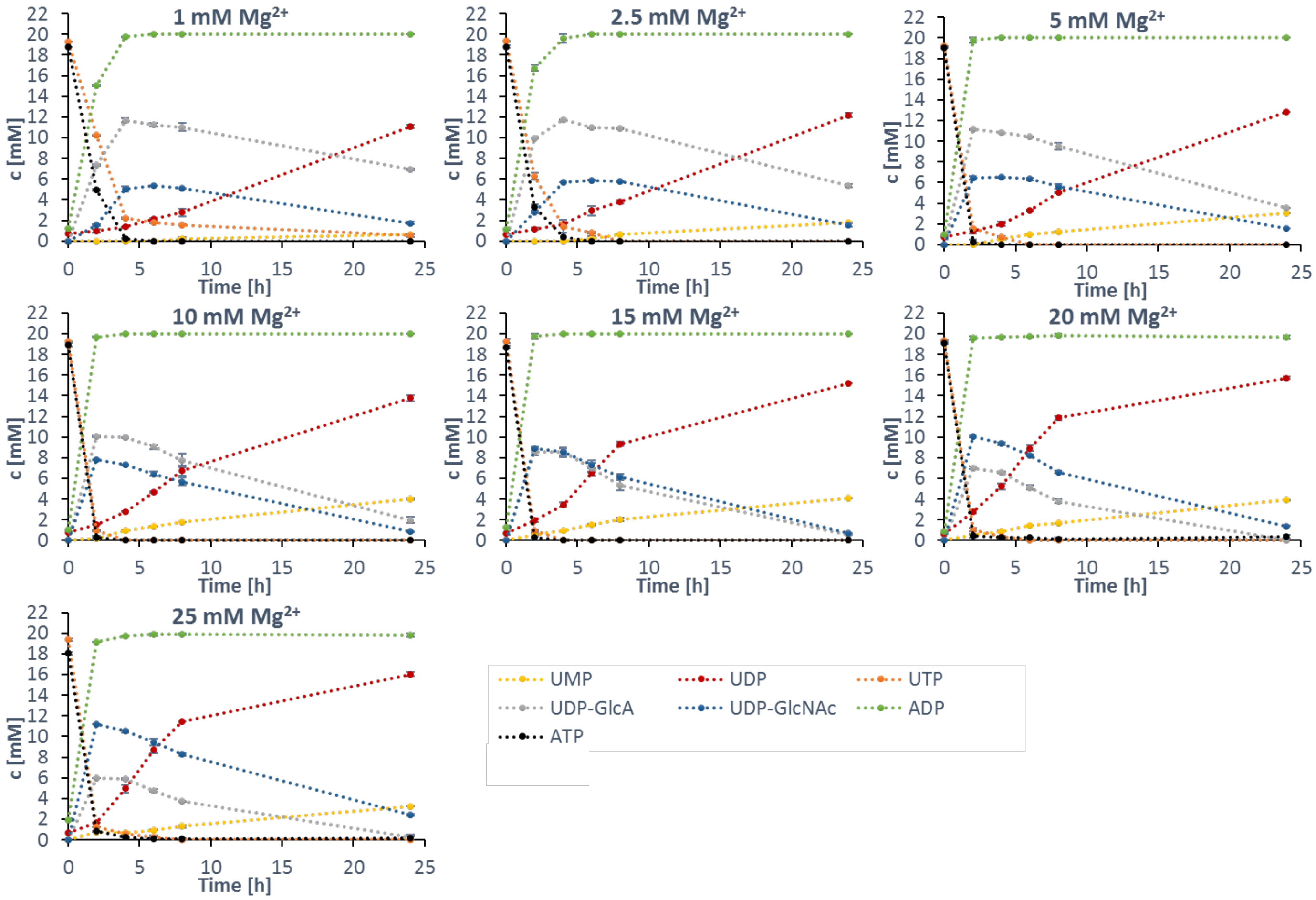

Figure S11. Controlling HA one-pot synthesis with Mg^{2+} concentration. 100 mM HEPES-NaOH pH 8, 25 °C, 10 mM GlcA, 10 mM GlcNAc, 20 mM ATP, 20 mM UTP, X mM Mg^{2+}, 10 mM K^{+}, 1.5 mM Mn^{2+}, 123.50 µg/mL AtGlcAK, 116.71 µg/mL AtUSP, 1202.42 µg/mL BlNahK, 254.63 µg/mL GlmU, 369.44 µg/mL PmPpA, 1289.14 µg/mL PmHAS. A volume of 5 mL was used. The reaction of specific Mg^{2+} concentrations was analysed with MP-CE over time.

Table S2. Regulation of the one-pot synthesis by Mg^{2+} concentration. HA size measurement with SEC-RALS/LALS. Samples were taken after 24 h.

Mg^{2+}	Mw	Mn	Q	Max	Max 75 %	Min 25 %	Min
1 mM	0.85	0.808	1.052	1.67	0.98	0.714	0.62
2.5 mM	0.95	0.897	1.059	1.85	1.057	0.771	0.61
5 mM	1.073	0.998	1.075	1.969	1.202	0.879	0.646
10 mM	1.215	1.118	1.087	2.067	1.401	0.967	0.712
15 mM	1.413	1.317	1.073	2.597	1.601	1.146	0.889
20 mM	1.358	1.238	1.097	1.942	1.602	1.126	0.665
25 mM	1.546	1.474	1.049	2.941	1.631	1.3	1.189
